# Apolipoprotein C3 and circulating mediators of preadipocyte proliferation in states of lipodystrophy

**DOI:** 10.1016/j.molmet.2022.101572

**Published:** 2022-08-11

**Authors:** Brandao Bruna Brasil, Sakaguchi Masaji, Batista, Thiago Martins, Hu Jiang, Nie Song, Schepmoes Athena A, BonDurant Lucas, Moreau François, Qian Wei-Jun, Kulkarni N. Rohit, Kahn, C. Ronald

**Affiliations:** 1 Section of Integrative Physiology and Metabolism, Joslin Diabetes Center, Harvard Medical School, Boston, MA, USA; 2 Department of Metabolic Medicine, Faculty of Life Sciences, Kumamoto University, Kumamoto, Japan; 3 Section on Islet Cell and Regenerative Biology, Joslin Diabetes Center, Dept. of Medicine, BIDMC, Harvard Stem Cell Institute, Harvard Medical School, Boston, MA, USA; 4 Biological Sciences Division, Pacific Northwest National Laboratory, Richland, Washington 99354, United States; 5 Alnylam Pharmaceuticals, Cambridge, MA, USA

**Keywords:** Adipogenesis, Inter-cellular crosstalk, Apolipoprotein, Metabolic syndrome, Circulating growth factor

## Abstract

Adipogenesis is a complex process controlled by intrinsic and extrinsic factors that regulate preadipocyte proliferation, adipogenic capacity and maturation of metabolic function. Here we show that insulin and IGF-1 receptors are essential for mature adipocyte survival and that deletion of both IR and IGF1R specifically in fat using a tamoxifen inducible-AdipoQ-Cre (Ai-DKO) leads to rapid and severe loss of adipocytes in all depots, associated with a metabolic syndrome characterized by hypertriglyceridemia, hyperglycemia, hyperinsulinemia, fatty liver, and pancreatic beta cell proliferation. In this model, this pathological phenotype reverses over a few weeks, in large part, due to preadipocyte proliferation and adipose tissue regeneration. Incubation of preadipocytes with serum from the Ai-DKO mice *in vitro* stimulates cell proliferation, and this effect can be mimicked by conditioned media from liver slices of Ai-DKO mice, but not by media of cultured Ai-DKO adipocytes, indicating a hepatic origin of the growth factor. Proteomic analysis of serum reveals apolipoprotein C3 (APOC3), a protein secreted by liver, as one of the most upregulated proteins in the Ai-DKO mice. *In vitro*, purified and delipidated APOC3 stimulates preadipocyte proliferation, however, knockdown of hepatic APOC3 *in vivo* in Ai-DKO mice is not sufficient to block adipose regeneration. Thus, lipodystrophy is associated with presence of increased preadipocyte-stimulating growth factors in serum. Our study indicates that APOC3 is one contributing factor to preadipocyte proliferation, however, other still-unidentified circulating growth factors are also likely present in Ai-DKO mice. Identification of these factors may provide a new approach to regulation of adipose mass in health and disease.

## Highlights

• Deletion of IR/IGF1R in adipocytes leads to lipodystrophy and metabolic syndrome.
• This is reversed due to preadipocyte proliferation and adipose tissue regeneration.
• Preadipocyte proliferation is induced by growth factors present in the circulation.
• APOC3, which is increased in these mice, contributes to preadipocyte proliferation.

## 1 Introduction

Regulation of adipose tissue mass and function is fundamental to whole-body metabolism. Excessive adipose tissue (obesity) or pathological fat loss (lipodystrophy) is often accompanied by metabolic disorders, including hyperlipidemia, hyperglycemia, insulin resistance and metabolic syndrome. These perturbations are secondary to the loss of the ability of adipose tissue to store fat, loss of normal adipose-derived hormonal secretions, as well as inflammation in the adipose tissue which may contribute to increased lipolysis and further ectopic lipid accumulation in non-fat tissues, including liver and skeletal muscle - all of which eventually lead to systemic insulin resistance and a wide range of metabolic abnormalities [31,43].

Previous studies from our lab [1,3,4,14,41] and others [52] have shown that adipogenesis and adipose tissue survival is blunted when insulin signaling is impaired. Consistent with this, chemical inhibition [52] or deletion of the insulin receptor specifically in adipocytes leads to severe lipodystrophy, associated with hepatosteatosis, insulin resistance accompanied by pancreatic β-cell proliferation and a metabolic syndrome phenotype [1,4,14,41]. Recently, we have shown that adipocyte loss and its associated metabolic changes can be minimized by simultaneous deletion of the three Forkhead box protein O present in fat (FOXO1, 3, and 4) indicating an autonomous mechanism of adipocyte survival and function dependent on IR, IGF1R, and their downstream signaling through FOXO transcription factors [14].

Interestingly, when IR or both IR/IGF1R are deleted in mature adipocytes of adult mice using an adipocyte-specific tamoxifen-inducible Cre, the resultant mice exhibit rapid adipose tissue loss and a similar metabolic syndrome, but in this case, the phenotype is transient, reaching its maximum at 3–6 days following completion of the tamoxifen treatment, after which there is a gradual reversal of these features over the following 10–30 days. The reason for this is an unexplained rapid regeneration of adipose tissue. Using an adipocyte-specific lineage-tracing mouse model we show that this process involves a robust proliferation of preadipocytes and their rapid differentiation into new mature adipocytes, over just a 30-day period [41]. The mechanism responsible for this remarkable adipose tissue growth is unknown.

Here we show that regeneration of adipose tissue, as well as the initial β-cell proliferation, are independent of hyperglycemia and not blocked by administration of a SGLT2 inhibitor. We find that serum of the inducible adipose-specific IR and IGF1R knockout mice (Ai-DKO mice) can stimulate preadipocyte, but not beta-cell, proliferation *in vitro*. LC-MS/MS based proteomics of the serum of Ai-DKO mice reveals significant upregulation of 107 proteins, of which apolipoprotein C3 is one of the most upregulated. We further demonstrate that purified APOC3 can induce preadipocyte proliferation *in vitro*. Liver specific knockdown of APOC3 in Ai-DKO mice *in vivo*, however, is not sufficient to block the regeneration of adipose tissue indicating that, in addition to APOC3, acute lipodystrophy induces other circulating factors which can stimulate adipose tissue regeneration *in vivo*.

## 2 Results

### 2.1 Adipose tissue-specific inducible deletion of insulin and IGF1 receptors leads to transient metabolic syndrome

**Figure 1:**
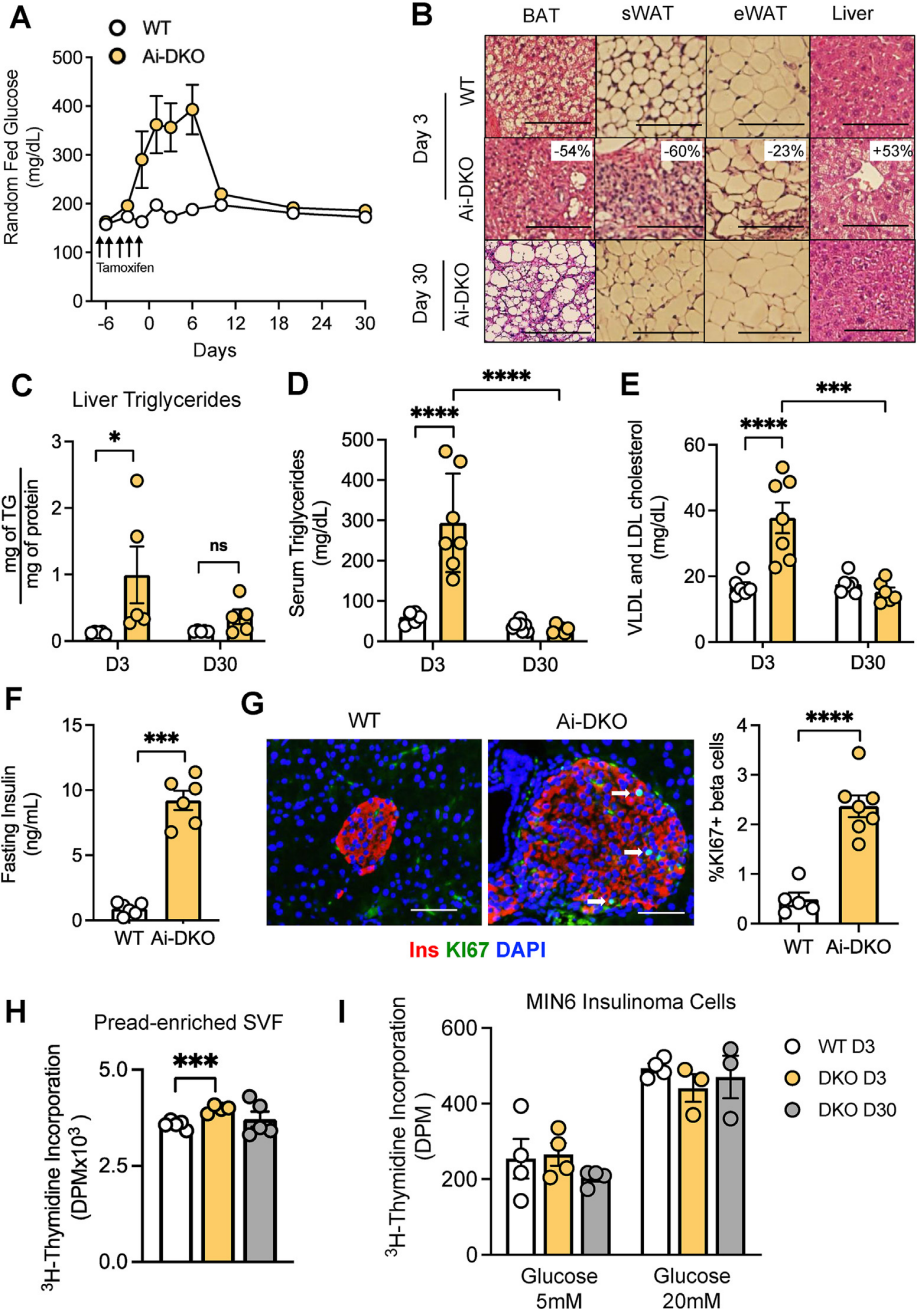
**Transient metabolic syndrome in Ai-DKO is associated with growth factors in the serum.** Eight-week-old, male, WT (IR f/f and IGF1R f/f) and Ai-DKO (Adiponectin-CreERT2; IR f/f and IGF1R f/f) were treated with 100 mg/kg tamoxifen by intraperitoneal injection five times over 6 days as indicated by arrows. The day of the last tamoxifen injection was considered experimental Day 0. (A) Random fed glucose over the course of 37 days. Glucose level was measured via tail vein blood using Infinity glucose meters (WT n = 7 and Ai-DKO n = 7). (B) H&E-stained sections of adipose tissue at Days 3 and 30. Scale bars, 50 μm (BAT = brown adipose tissue; sWAT = subcutaneous white adipose tissue and eWAT = epididymal white adipose tissue). (C) Liver triglycerides assessed at Days 3 (D3) and 30 (D30) in WT (n = 5) and Ai-DKO (n = 5) mice. (D) Fasting serum triglycerides assessed at Days 3 (D3) and 30 (D30). For WT mice at D3 n = 6 and at D30 n = 8; for Ai-DKO mice at D3 n = 7 and at D30 n = 6. (E) Fasting VLDL and LDL cholesterol assessed at Days 3 (D3) and 30(030). WT (D3 n = 6 and D30 n = 6); for Ai-DKO mice (D3 n = 7 and D30 n = 6). (F) Fasting insulin assessed at Day 3 (D3) (WT n = 6 and Ai-DKO n = 6), (G) Pancreatic sections immunostained for insulin and Ki67 in WT and Ai-DKO at Day 3 after tamoxifen treatment. Scale bars, 100 μm (left panel). Quantification of Ki67+ insulin + cells in control pancreas sections at Day 3 (right panel). WT n = 5 and Ai-DKO n = 7. (H) ^3^H-thymidine incorporation in preadipocyte-enriched SVF (stromal vascular fraction) from subcutaneous WAT treated with 10% of serum from WT mice Day 3 (D3) and Ai-DKO mice Days 3 and 30 (D3 and D30) for 48 h (n = 5 experimental replicates). This experiment was performed 3×. (I) ^3^H-thymidine incorporation in MIN6 treated with 10% of serum from WT mice Day 3 (D3) and Ai-DKO mice Days 3 and 30 (D3 and D30) for 72 h. Glucose concentrations were 5 or 20 mM. (n = 3–5 experimental replicates) This experiment was performed 2×. Data were analyzed using a t-test, 1-way ANOVA with repeated or 2-way ANOVA as appropriate. ∗p < 0.05, ∗∗∗p < 0.001 and ∗∗∗∗p < 0.0001. Bars represent ±SEM. (For interpretation of the references to color in this figure legend, the reader is referred to the Web version of this article.)

We have previously shown that insulin and IGF1 receptors are essential to the maintenance and survival of adipose tissue [4,14,41]. Constitutive adipose tissue-specific deletion of IR and IGF1R using an aP2 promoter Cre, and even more so using an adiponectin promoter Cre, leads to a marked lipodystrophy phenotype with almost complete loss of both white and brown adipose tissue accompanied by marked glucose intolerance, fatty liver, hyperlipidemia, hyperinsulinemia with islet hyperplasia and insulin resistance [1,4,14,46]. Corroborating our previous findings [41], inducible deletion of IR and IGF1R in mature adipocytes of adult mice using an adiponectin-Cre^ERT2^ also resulted in rapid development of lipodystrophy and metabolic syndrome, but in this case the metabolic features were transient (Figure 1(A) and (B)). Thus, the fat-specific inducible IR/IGF1R knockout (Ai-DKO) mice exhibited a marked elevation in circulating glucose level as early as Day 4 of in the 5-day course of tamoxifen injections, and this continued to increase for 3–6 days after the last tamoxifen injection, after which glucose levels declined and returned to near control levels by day 9–12 (Figure 1(A)). The onset of these changes at Day 3 was associated with an ∼54% decrease in the mass of intrascapular brown fat, an ∼60% decrease in subcutaneous inguinal white fat and an ∼23% decrease in visceral/perigonadal white adipose tissue, in each case associated with a loss of intracellular lipid (Figure 1(B) and Supplement Figure 1(A)). This was also accompanied by ∼2.6-fold increase in circulating free fatty acids (FFA) (Supplement Figure 1(B)), ectopic accumulation of lipid in the liver with an ∼53% increase in liver weight, a 7.8-fold increase in liver triglyceride content (Supplement Figure 1(A) and Figure 1(C)), a ∼5-fold increase in serum triglycerides and a 2.2-fold increase in VLDL/LDL cholesterol (Figure 1(D)–(E)). Mice also developed insulin resistance as manifested by a 32% increase in fasting glucose levels (Supplement Figure 1(C)) and an almost 10-fold increase in fasting plasma insulin levels (Figure 1(F)). In addition, Ai-DKO mice exhibited a significant increase in beta-cell proliferation manifested by a more than doubling in islet size, a 5-fold increase in Ki67 staining in beta-cells. Similarly, we observed a significant upregulation of genes involved in cell cycle regulation, including Aurora A, cyclins A2, B, E1/2, F, and cyclin dependent kinase 1 (Cdk1) in isolated pancreatic beta-cell (Figure 1(G) and Supplement Figure 1(E)). In this inducible model, however, the hyperglycemic hyperinsulinemic metabolic syndrome phenotype was spontaneously almost completely reversed by 30 days after the last tamoxifen injection (Figure 1(A) and 1(C)–(E)). This was also accompanied by a restoration of lipid droplet size in all three fat depots and a regain of white adipose tissue mass, as well as reversal of hepatosteatosis and hepatomegaly (Supplement Figure 1(A) and Figure 1(B)). No changes were observed in serum HDL (Supplement Figure 1(D)).

The phenomenon of adipocyte regeneration and β-cell proliferation led us to hypothesize the existence of a circulating growth factor or factors that could lead to proliferation in both cell types. To begin to identify the putative adipocyte growth factor, we isolated the preadipocyte-enriched stromal vascular fraction (SVF) from the subcutaneous white adipose tissue of wild-type mice, expanded these cells in culture for 5 days, serum-starved the cells for 6–12 h, and then incubated it for 48 h with serum from wild type or Ai-DKO mice collected at Day 3 and Day 30. We observed that Day 3 Ai-DKO serum significantly induced preadipocyte proliferation, as measured by ^3^H-thymidine incorporation (Figure 1(H)), and this effect was almost completely lost in Day 30 serum (Figure 1(H)). To determine if the serum from Ai-DKO would also induce beta-cell proliferation, MIN6 mouse insulinoma cells were serum starved for 6 h then treated with serum from WT or Ai-DKO from Days 3 and 30 at two different glucose concentrations for 72 h. While beta-cell proliferation was clearly enhanced by hyperglycemia, it was not significantly altered by the Ai-DKO serum (Figure 1(I)), suggesting that preadipocyte proliferation and beta-cell growth are regulated by different factors or different combinations of factors.

### 2.2 The role of hyperglycemia in lipodystrophy and the metabolic syndrome

**Figure 2:**
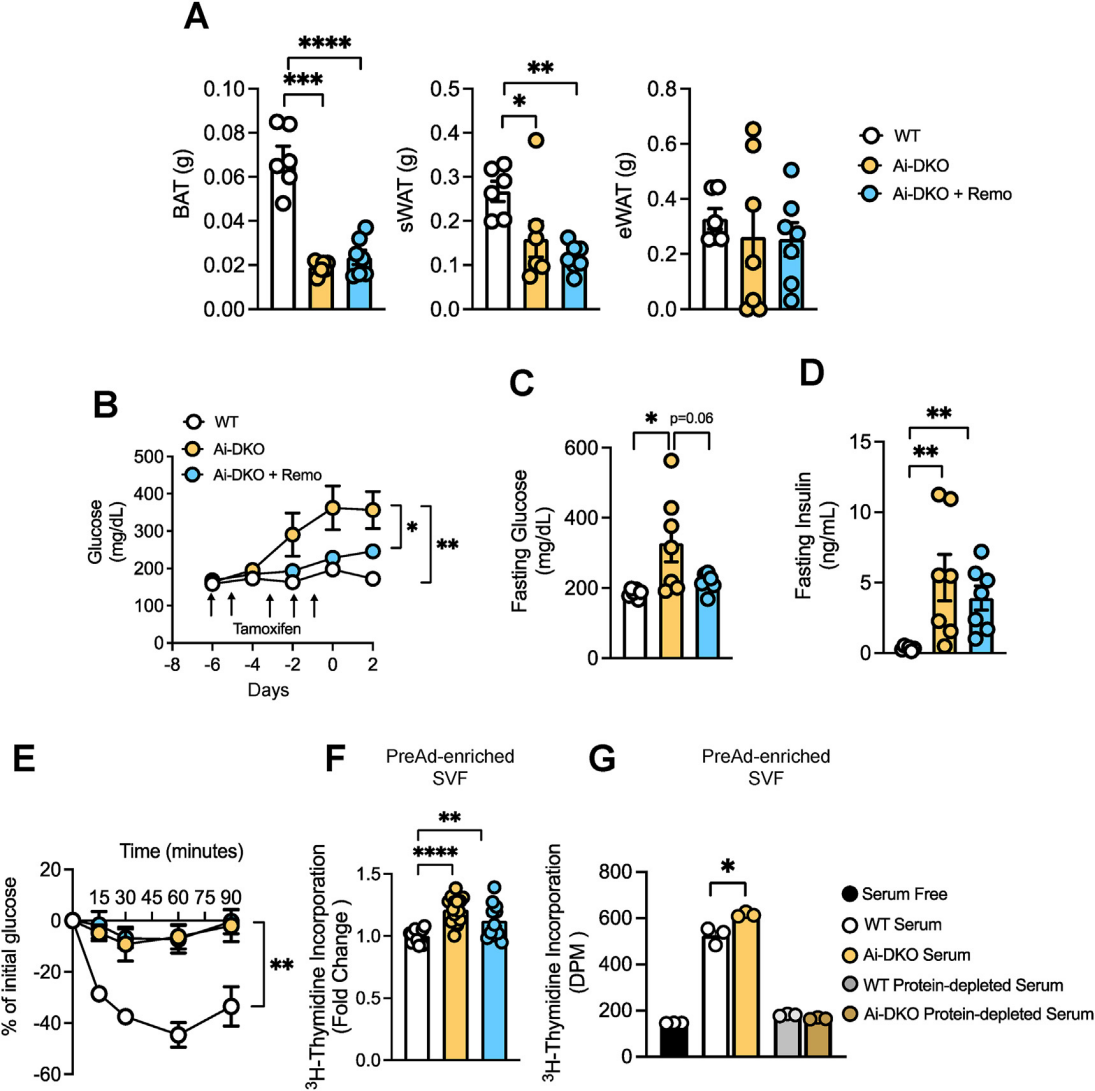
**Preadipocyte proliferation is induced independent of hyperglycemia.** Eight-week-old, male WT (IR f/f and IGF1R f/f) and Ai-DKO (Adiponectin-CreERT2; IR f/f and IGF1R f/f) mice were treated with 100 mg/kg tamoxifen by intraperitoneal injection five times over 6 days. For one group Chow diet was supplemented with 0.03% of remogliflozin (Ai-DKO + Remo). The day of the last tamoxifen injection was considered experimental Day 0. (A) Adipose tissue weight (BAT = brown adipose tissue; sWAT = subcutaneous white adipose tissue and eWAT = epididymal white adipose tissue). Results are from WT n = 6, Ai-DKO n = 7 and Ai-DKO + Remo n = 7. (B) Random fed glucose levels over the course of 9 days measured using tail vein blood and Infinity glucose meters (WT n = 6, Ai-DKO n = 7 and Ai-DKO + Remo n = 7). (C) Fasting glucose assessed at Day 2 after the last tamoxifen injection measured as above (WT n = 6, Ai-DKO n = 7 and Ai-DKO + Remo n = 7). (D) Fasting insulin assessed at Day 2 after the last tamoxifen injection (WT n = 6, Ai-DKO n = 7 and Ai-DKO + Remo n = 7). (E) Insulin tolerance test (ITT) performed at Day 2 after the last tamoxifen injection. Glucose was measured via tail vein blood using Infinity glucose meters (WT n = 6, Ai-DKO n = 7 and Ai-DKO + Remo n = 7). (F) ^3^H-Thymidine incorporation in preadipocyte-enriched SVF (stromal vascular fraction) from sWAT treated with 10% of serum from Day 3 WT, Ai-DKO and Ai-DKO + Remo mice for 48 h (n = 4–5 experimental replicates). This experiment was performed 3×. (G) ^3^H-Thymidine incorporation in preadipocyte-enriched SVF (stromal vascular fraction) from subcutaneous WAT treated with serum-free media, 10% serum or protein-depleted serum from WT or Ai-DKO from Day 3. Cells were incubated for 48 h with (n = 3 experimental replicates). Data were analyzed using t-test, 1-way ANOVA or 1-way ANOVA with repeated measures as appropriate. ∗p < 0.05, ∗∗p < 0.01, and ∗∗∗∗p < 0.0001. Bars are expressed as ±SEM. (For interpretation of the references to color in this figure legend, the reader is referred to the Web version of this article.)

To determine to what extent hyperglycemia contributes to the transient lipodystrophy and metabolic syndrome in Ai-DKO mice, we treated mice with the SGLT2 inhibitor remogliflozin (Remo). This was mixed into the diet beginning one week prior the tamoxifen treatment and continued until 3 days after completion of the induction of recombination (Day 3). While Remo did not affect overall body weight (Supplement Figure 2(A)) or adipose tissue loss due to IR/IGF1R knockout (Figure 2(A)), Remo treatment did prevent the development of random fed and fasting hyperglycemia in the Ai-DKO mice (Figure 2(B) and (C)). This was not sufficient, however, to prevent the development of insulin resistance or the associated hyperinsulinemia which accompanied the lipodystrophy in Ai-DKO mice (Figure 2(D) and (E)). Likewise, SGLT2 inhibition did not prevent the development of hepatomegaly or the increase in triglyceride accumulation in liver of the Ai-DKO mice (Supplement Figure 2(B) and (C)).

To determine if normalizing blood glucose in the Ai-DKO would affect the presence of the circulating factor capable of stimulating preadipocyte proliferation, we isolated primary preadipocytes from wild type mice and cultured them for 48 h with serum from wild type or Ai-DKO mice treated or not with remogliflozin. Again, we observed a significant increase in proliferation after adding serum from Ai-DKO as compared to WT mice (Figure 2(F)). Although, this effect was slightly reduced in the REMO group, the cell proliferation effect of Ai-DKO serum remained significant compared to WT indicating that, although hyperglycemia might contribute somewhat to the levels or activity of the preadipocyte-proliferation factor(s), proliferation was induced by a factor or factors that were largely independent of glucose.

To further investigate if the cell proliferation was driven by proteins versus a lower molecular weight metabolite, serum from WT and Ai-DKO mice was filtered using a membrane with 3 kDa cutoff, and the flow-through (protein-depleted) was used to stimulate the cells. As expected, unfiltered serum from WT mice induced an ∼3.5-fold increase in proliferation of preadipocytes, as measured by ^3^H-thymidine incorporation, as compared to basal rates in cells which had been serum starved, and this was increased to ∼4.2-fold using serum from Ai-DKO mice. These effects were completely lost in the protein-depleted serum (Figure 2(G)), indicating that a molecule or molecules larger than 3 kDa, likely a protein, are the drivers of proliferation in serum.

### 2.3 An in vitro model of acquired lipodystrophy

Mature adipocytes are known to secrete a wide range of molecules that regulate systemic metabolism through their action on other cells throughout the body [8,25,50]. To investigate whether the adipotrophic factor in Ai-DKO mice was directly derived from adipocytes, we isolated the stromal vascular fraction from subcutaneous white adipose tissue of IR/IGF1R floxed Adiponectin-CreERT2 positive mice and immortalized the preadipocytes using retroviral SV40 large T antigen [44]. These cells then underwent differentiation using a standard 8-days protocol, after which the cells were treated with either vehicle (ethanol = Flox) or 10 μM of 4-hydroxytamoxifen to induce recombination (i-DKO). The culture media was then changed to DMEM-High glucose supplemented with 10% FBS for 24 h, after which fresh media was added for 48 h. This final media was collected and considered conditioned media (CM).

**Figure 3:**
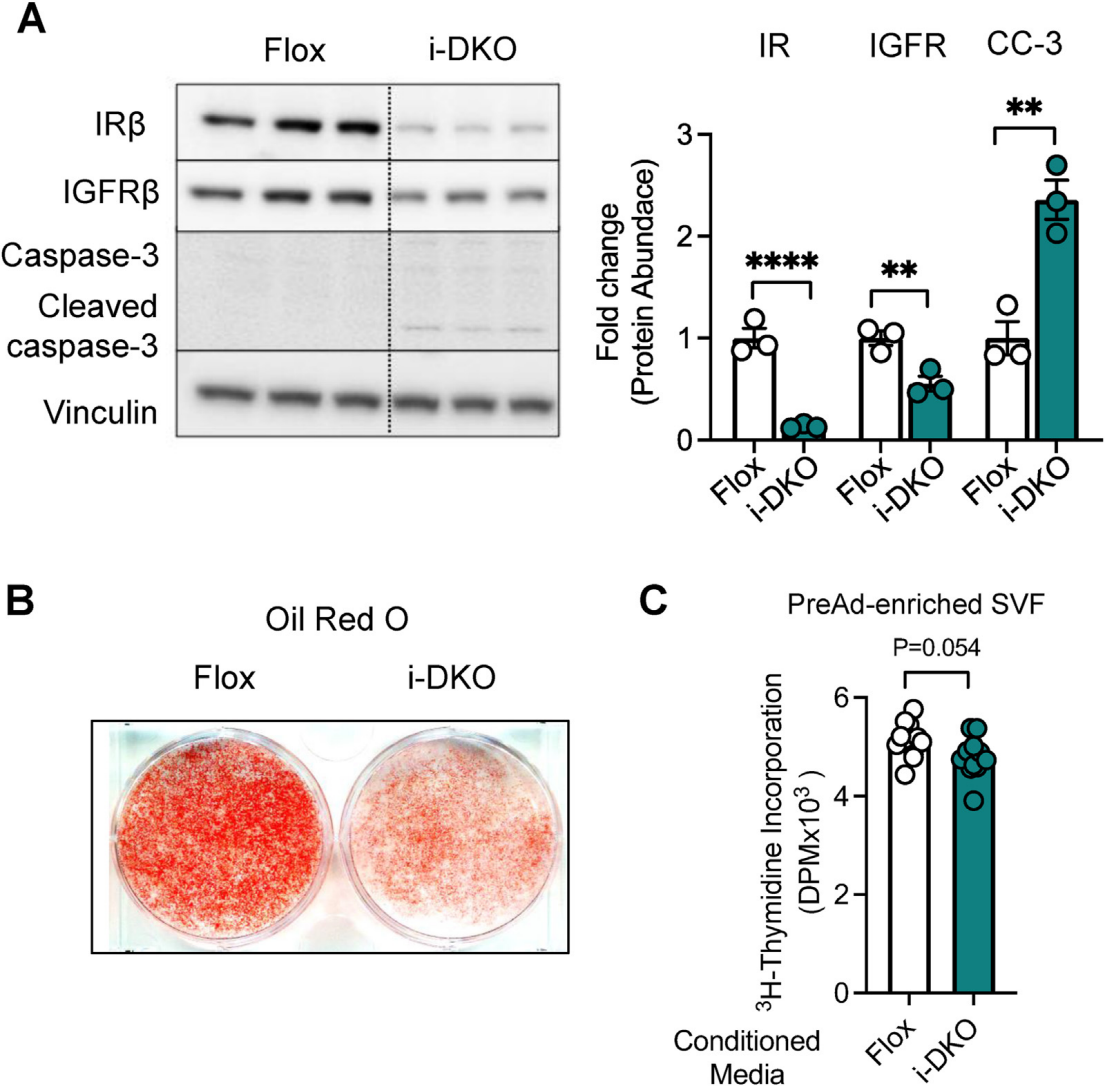
**Characterization of inducible-IR/IGF1R knockout adipocytes (i-DKO) and proliferative potential of i-DKO-derived conditioned media.** The stromal vascular fraction from subcutaneous white adipose tissue from Adipoctin-CreERT2; IRf/f and IGF1Rf/f mice was isolated and immortalized using retroviral SV40T and stimulated to undergo differentiation as in Appendix A. After adipocyte differentiation, cells were treated with either with vehicle (ethanol) or 10 μM of 4-OH-tamoxifen to induce recombination (i-DKO) and then studied 48 h later. (A) Western blot (left) and densitometric quantification (right) of Insulin and IGF-1 Receptor β-subunit (IR-β, IGF1Rβ), Caspase-3, and cleaved caspase-3 (CC-3) in Floxed and Inducible-IR/IGFR knockout adipocytes (i-DKO). Protein concentrations were normalized to vinculin (n = 3 experimental replicates.). (B) Oil Red O staining of Floxed and Inducible-IR/IGF1R Knockout adipocytes (i-DKO). (C) ^3^H-Thymidine incorporation in preadipocyte-enriched SVF (stromal vascular fraction) from sWAT treated with conditioned media (CM) from Floxed or i-DKO adipocyte cells for 48 h (n = 10 experimental replicates_ This experiment was performed 1×). Data were analyzed using t-test. ∗∗p < 0.01 and ∗∗∗∗p < 0.0001. Bars represent as ±SEM. (For interpretation of the references to color in this figure legend, the reader is referred to the Web version of this article.)

We found that i-DKO cells *in vitro* mimicked the phenotype of adipocytes *in vivo* ([41] and this study). Thus, following tamoxifen treatment there was an ∼80% decrease in IR protein levels and a somewhat lesser decrease in IGF1R protein, as determined by western blotting (Figure 3(A)). Compared with floxed cells, these i-DKO cells also exhibited a major decrease in lipid content as demonstrated by Oil Red O staining (Figure 3(B)). In addition, there was an increase in the abundance of cleaved caspase 3, a cell marker for apoptosis (Figure 3(A)). When primary PreAds from a WT mouse were incubated with conditioned media from WT or i-DKO adipocytes for 48 h, we observed a tendency to decreased cell proliferation, rather than the increase in proliferation observed in cells treated with Ai-DKO serum (Figure 3(C)), suggesting that i-DKO-adipocytes were not themselves the source of the preadipocyte growth factor.

### 2.4 Serum protein composition is significantly altered in Ai-DKO mice

**Figure 4:**
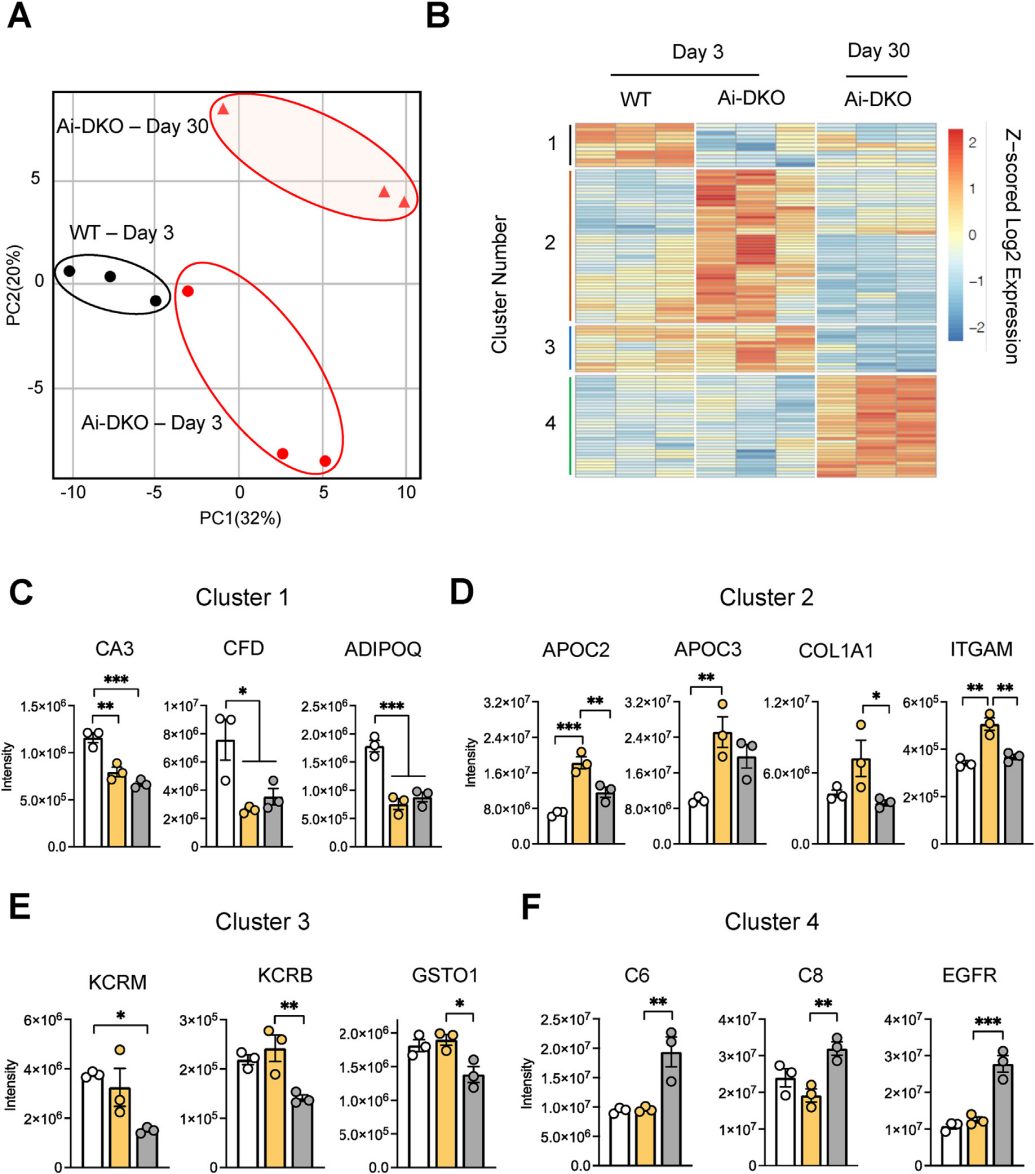
**WT and Ai-DKO serum proteome assessed by LC-MS/MS on Day 3 and Day 30.** Serum was obtained from 6 h fasted mice at Day 3 and Day 30 after completion of tamoxifen injection. The serum protein profile was determined by LC-MS/MS as described in Appendix A. (A) Principal-component analysis (PCA) and (B) hierarchical clustering analysis of the serum protein abundance data from WT on Day 3 and Ai-KO on Days 3 and 30 (n = 3). (C–F) Examples of proteins present on Clusters 1, 2, 3 and 4, respectively (n = 3). Data were analyzed using Moderated F-tests and hierarchical clustering. ∗p < 0.05, ∗∗p < 0.01, and ∗∗∗p < 0.001. Bars represent the ±SEM.

To perform an unbiased search for potential circulating preadipocyte growth factors, we collected the serum of WT and Ai-DKO mice at Day 3 after tamoxifen (the peak of fat loss and metabolic syndrome) and Day 30 (after recovery of most of the lost fat) and performed proteomics using liquid chromatography and tandem mass spectrometry (LC-MS/MS) [32]. An average of 1660 proteins were detected in all samples. Principal component analysis (PCA) revealed three distinct protein groups (Figure 4(A)) based on abundance in WT and Ai-DKO on Days 3 and 30. Comparison of proteins that were significantly different in levels in Ai-DKO on Day 3 or Day 30 compared to WT, at a FDR< 0.05 and with an absolute fold change of >│1.25│, revealed 107 differentially regulated proteins (Supplement Table 1). A heat map showing the changed proteins with hierarchical clustering revealed four different clusters (Figure 4(B)). The first cluster was composed by 11 proteins that were down-regulated in Ai-DKO mice at Day 3, some of which recovered by Day 30 and some of which did not. The second cluster was composed of 49 proteins that were upregulated in Ai-DKO at Day 3, most of which returned to near normal by Day 30. Clusters 3 and 4 were composed of 47 proteins, of which 15 were down and 32 were upregulated, but only at Day 30. Cluster 1 included two well-known adipokines, adipsin (complement factor D or CFD) [39] and adiponectin (ADIPOQ) [54] and the adipocyte-enriched protein carbonic anhydrase 3 (CA3) (Figure 4(C)). Cluster 2 contained several apolipoproteins, including apolipoproteins C2, C3 and C4 (APOC2, APOC3 and APOC4), as well as several matrix proteins, such as collagen alpha-1(I) chain (COL1A1) and integrin alpha-M (ITGAM). Cluster 3 included creatine kinase M and B isoforms, as well as glutathione S-transferase omega-1 (GSTO1) (Figure 4(E)). Finally, among the upregulated proteins in cluster 4 were complement proteins C6 and C8 and epidermal growth factor receptor (EGFR) (Figure 4(F)).

We focused on the proteins that were upregulated on Day 3 and not completely reversed on Day 30 as candidate preadipocyte growth factors, since serum from Ai-DKO mice induced proliferation by Day 3, and this tendency continued to Day 30 (Figure 1(H)). This highlighted the apolipoproteins, especially APOC3, as potential inducers of preadipocyte proliferation. Indeed, APOC3 was the most upregulated protein in the serum of Ai-DKO mice on Day 3 at 2.7-fold and remained elevated at 1.9-fold on Day 30 (Figure 4(D)). Furthermore, the increased circulating APOC3 was accompanied by upregulation of its protein levels in the liver of Ai-DKO mice (Supplement Figure 3(A)).

### 2.5 Apolipoprotein C3 induces preadipocyte proliferation in vitro

**Figure 5:**
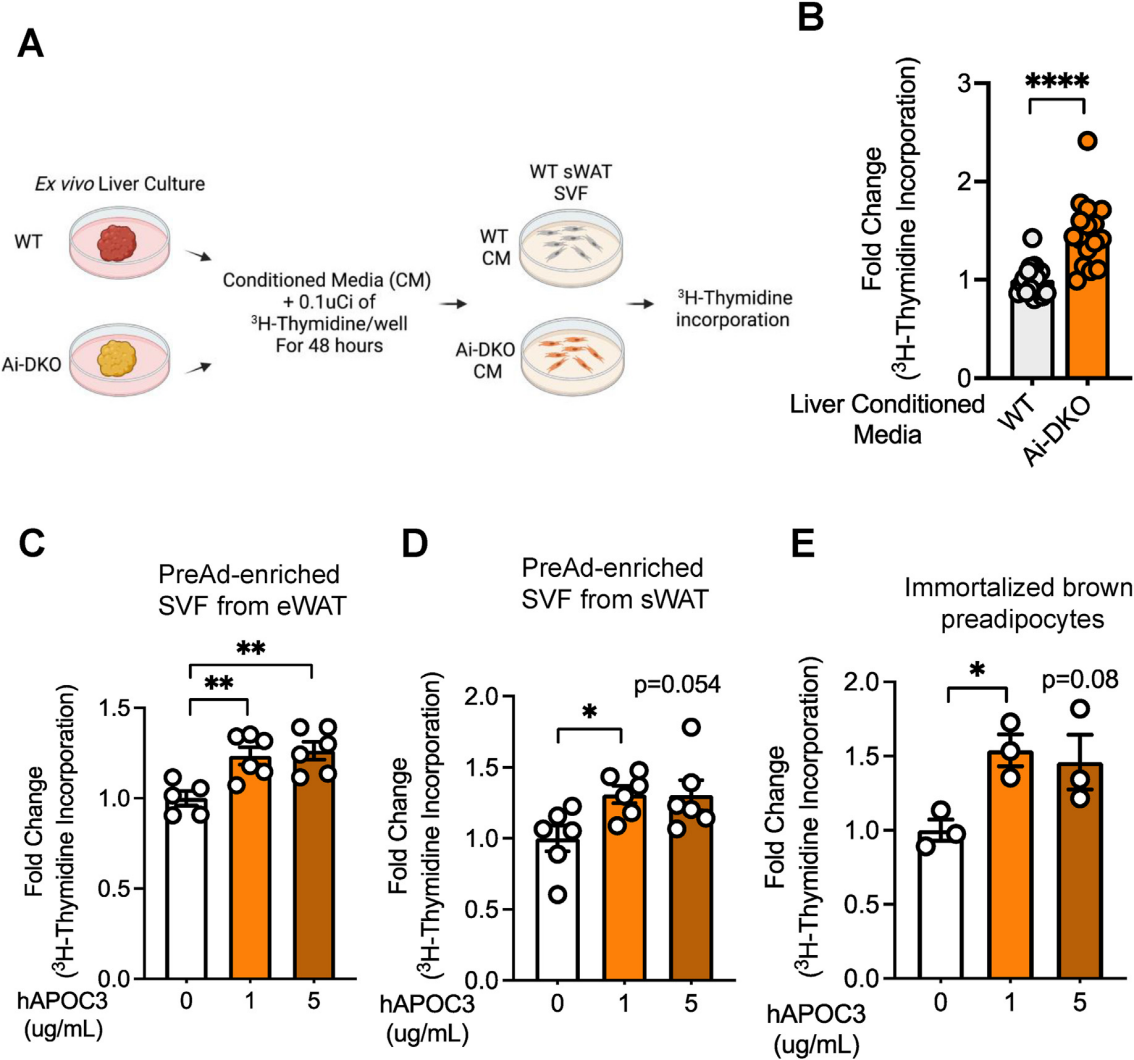
**Apolipoprotein C3-indued preadipocyte proliferation.** (A) Experimental design for the *ex vivo* liver culture and proliferation assay. On Day 3 after the last tamoxifen injection, an ∼100 mg liver slice was taken from WT and Ai-DKO mice, washed twice with PBS, and then cultured in 1 ml of serum-free Dulbecco's modified Eagle's medium (DMEM) with 5.5 mM glucose for 6 h at 37 °C, after which the conditioned medium was collected, centrifuged, and used to stimulate the preadipocyte-enriched SVF from the subcutaneous white adipose tissue for 48 h. (B) ^3^H-Thymidine incorporation in preadipocyte-enriched SVF from sWAT treated with conditioned media (CM) from WT or Ai-DKO *ex vivo* liver culture (WT n = 14 and Ai-DKO n = 16 experimental replicates). This experiment was performed 2×. (C–D) ^3^H-Thymidine incorporation in preadipocyte-enriched SVF from sWAT, eWAT and immortalized brown adipocytes treated with 1 or 5 μg/ml of purified lipid-free human apolipoprotein C3 (hAPOC3 – 16-16-120303-L) for 48 h (For sWAT-hAPOC3 0, 1 and 5 μg/ml (n = 5, 6 and 6); for eWAT-hAPOC3 0, 1 and 5 μg/ml (n = 6, 6 and 5); for BAT-hAPOC3 0, 1 and 5 μg/ml (n = 3). This experiment was performed 3×. Data were analyzed using t-test. ∗p < 0.05, ∗∗p < 0.01, and ∗∗∗∗p < 0.0001. Bars represent the ±SEM. . (For interpretation of the references to color in this figure legend, the reader is referred to the Web version of this article.)

Since Apolipoprotein C3 is primarily secreted from hepatocytes, to determine if liver was the main source of the preadipocyte (PreAd) proliferation factor, we performed an *ex-vivo* liver slice culture using livers from WT and Ai-DKO mice. In agreement with the *in vivo* data, APOC3 abundance was increased by 2-fold in the conditioned media derived from Ai-DKO liver compared to WT liver (Supplement Figure 4(A)). Importantly, when the conditioned media from WT and Ai-DKO *ex vivo* liver cultures was incubated with preadipocytes derived from subcutaneous adipose tissue SVF, we observed a significant ∼47% increase in PreAd proliferation with media from Ai-DKO liver vs WT liver (Figure 5(A) and (B)). Moreover, incubation of PreAds with conditioned media that had been pre-incubated with antibody against APOC3 blocked the proliferative effect of the Ai-DKO conditioned medium (Supplement Figure 4(B)). Most importantly, treatment of preadipocytes with human purified APOC3 (1 and 5 μg/ml), which had been delipidated, was also sufficient to induce proliferation in preadipocytes derived from eWAT and sWAT, indicating this effect was due to the APOC3 protein and not the lipid associated with it (Figure 5(C)–(D)). A similar effect of purified APOC3 on cell proliferation was observed with PreAds derived from the brown adipose tissue (Figure 5(E)).

### 2.6 APOC3 silencing in vivo improves hypertriglyceridemia but is not sufficient to block preadipocyte proliferation in vivo

**Figure 6:**
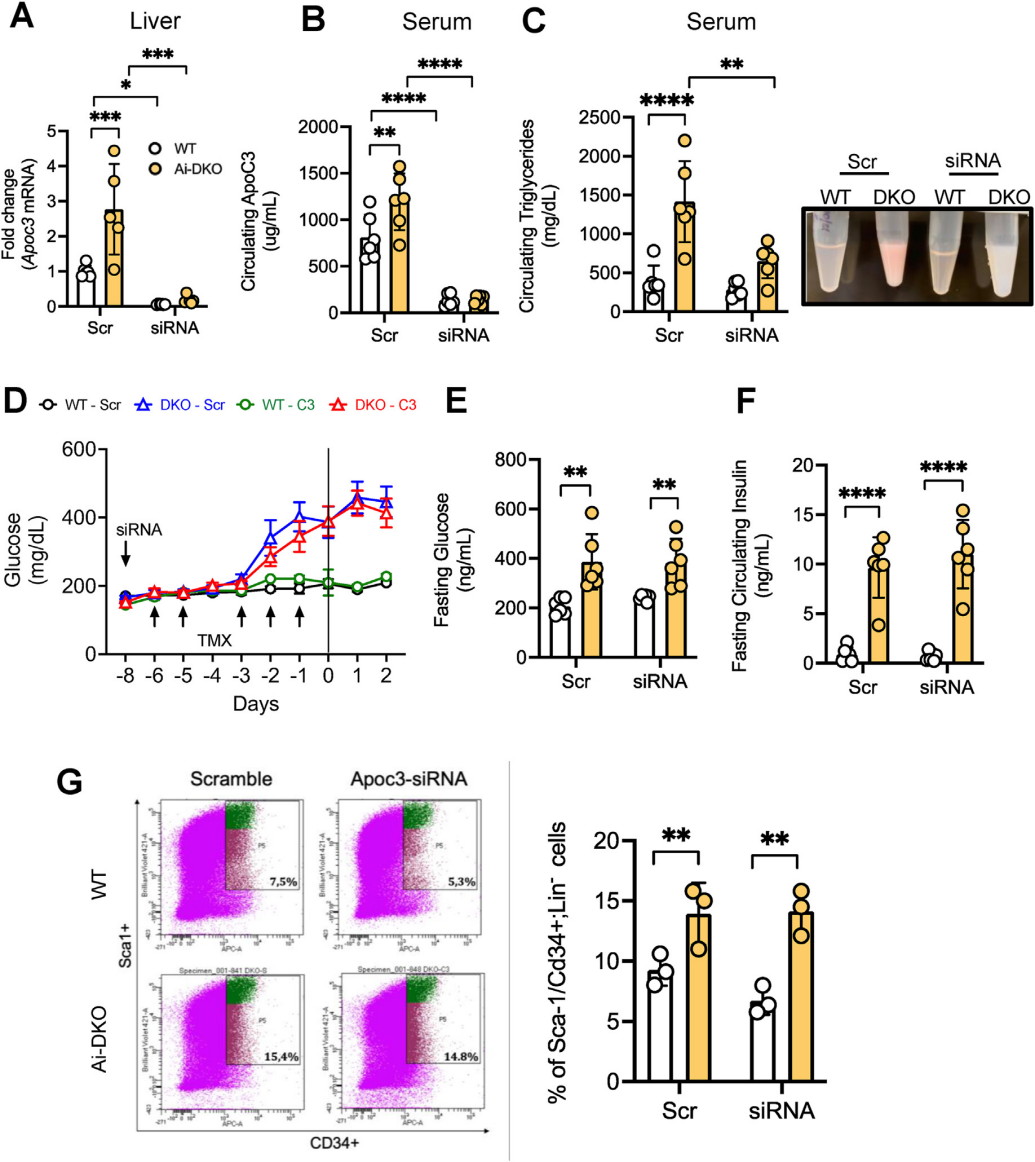
**Metabolic and cellular effects of Liver APOC3 knockdown.** 10-12 weeks-old, male IR f/f and IGF1R f/f mice and Ai-DKO (Adiponectin-CreERT2; IR f/f and IGF1R f/f) mice were given a single subcutaneous injection of APOC3 siRNA (10 mg/kg diluted in PBS) or scrambled control 2 days prior to the first intraperitoneal tamoxifen injection, then subjected to tamoxifen recombination as described in Appendix A. Mice were sacrificed on Day 3. N = 6 in all experiments, unless otherwise indicated. WT Scr and Ai-DKO Scr (WT and Ai-DKO treated with scrambled sequence) and WT siRNA and Ai-DKO siRNA (WT and Ai-DKO treated with siRNA targeting liver APOC3). (A) Liver *Apoc3* mRNA expression at Day 3 (B) Serum APOC3 level in random fed mice assessed as described in Appendix A (C) Random fed serum triglycerides (left) and representative image of serum appearance (right) of WT and Ai-DKO, scramble (Scr) and APOC3 siRNA (siRNA) treated mice at Day 3 (D3). (D) Random fed glucose over the course of 11 days during siRNA experiment. Glucose was measured via tail vein blood using Infinity glucose meters (WT-Scr = WT Scramble; Ai-DKO-Scr = Ai-DKO scramble; WT-C3 = WT APOC3 siRNA and Ai-DKO-C3 = Ai-DKO APOC3 siRNA.). (E) Fasting glucose at Day 3 was measured using tail vein blood as above. (F) Fasting serum insulin levels at Day 3 assessed as Appendix A. (G) FACS profiles of sorted subcutaneous white adipose-SVF at Day 3 from control mice (n = 3). Live cells of the lineage negative (Lin−) population, i.e., lacking CD31, CD45, and Ter119 expression, were considered preadipocytes based on positive Sca1 and CD34 expression. Representative data are indicated in the FACS profiles (left) and quantification of % Sca1/CD34+ cells is shown on the right. Data was analyzed using a 2-way ANOVA and 2-way ANOVA with repeated measurement as appropriated. ∗p < 0.05, ∗∗p < 0.01, ∗∗∗p < 0.001 and ∗∗∗∗p < 0.0001. Bars represent ±SEM.

To determine if APOC3 was the dominant factor involved in preadipocyte proliferation *in vivo*, we knocked-down this apolipoprotein by treating mice with small interfering RNA (siRNA) directed to APOC3 using a scrambled siRNA as a control. Two days later, tamoxifen induction of IR/IGF1R deletion in adipose tissue was initiated as described previously, and mice were sacrificed at Day 3 after the last tamoxifen injection, i.e., 11 days after the siRNA injection (Supplement Figure 5(A)). *Apoc3* gene expression and protein level were upregulated in the liver of Ai-DKO mice by ∼2.8-fold (Fig .6 A) and ∼2.3-fold respectively (Supplement Figure 5(B)). As expected, siRNA treatment reduced hepatic *Apoc3* expression by more than 90% in both groups (Figure 6(A)) and reduced hepatic APOC3 protein abundance by 30% in the Ai-DKO mice (Supplement Figure 5(B)). More importantly, circulating levels of APOC3, which were elevated by ∼47% in Ai-DKO mice, decreased by more than 90% in both genotypes (Figure 6(B)). This had no effect on the expression of intestinal (jejunum) *Apoc3* (Supplement Figure 5(C)), confirming the tissue specificity of the siRNA. Liver APOC3 knockdown did not affect the development of the lipodystrophy syndrome (Supplement Figure 5(D)), overall body weight (Supplement Figure 5(E)), or development of hyperglycemia, hyperinsulinemia (Figure 6(D)–(F)) or glucose intolerance in Ai-DKO mice (Supplement Figure 5(E)). Also, there was no significant effect to reduce hepatomegaly or liver triglyceride content (Supplement Figure 5(D) and (G)), and we still observed a significant increase in the pancreatic islet area suggesting that the mechanisms leading to beta-cell proliferation in Ai-DKO mice were still intact (Supplement Figure 5(I)). On the other hand, APOC3 knockdown, did result in ∼50% reduction in the excess levels of circulating triglycerides in Ai-DKO mice (Figure 6(C)). In addition, the expression of the lipogenic enzymes *Acaca* (Acetyl-Coenzyme A carboxylase alpha 1) and *Fasn* (Fatty acid synthase) was significantly reduced in the liver of Ai-DKO with APOC3 knockdown when compared to Ai-DKO mice treated with Scr control (Supplement Figure 5(H)). Expression of *Scd1* (stearoyl-Coenzyme A desaturase 1), on the other hand, which was significantly increased in Ai-DKO, was not affected by APOC3 knockdown (Supplement Figure 5(H)).

To determine whether knockdown of APOC3 could regulate preadipocyte proliferation *in vivo*, we performed fluorescence-activated cell sorting (FACS) analysis of the stromal vascular fractions (SVFs) from subcutaneous WAT of control and Ai-DKO mice at Day 3 and quantified the number of preadipocytes using established preadipocyte markers (Lin− Sca1+ CD34+) [5]. Consistent with increased preadipocyte proliferation, we observed a more than doubling in the percentage of PreAds in the SVF in the Ai-DKO mice, increasing from 7.5% ± 1.0% to 15.4% ± 2.2% (p < 0.01). However, similar increase in preadipocyte percentage was observed in Ai-DKO with an APOC3 knockdown (Figure 6(G)). Thus, while APOC3 can act as a preadipocyte stimulating growth factor *in vitro*, *in vivo* APOC3 knockdown is not sufficient to inhibit preadipocyte proliferation, indicating that APOC3 acts with other factors to produce this regenerative response.

## 3 Discussion

Insulin and IGF-1 receptors are fundamental for adipose tissue function and survival. Here we show that an adipose specific inducible deletion of IR and IGF1R (Ai-DKO) leads to a rapid loss of white and brown adipose tissue. This is associated with uncontrolled lipolysis and increased loss of adipocytes due to apoptosis resulting in lipid accumulation in the liver, impaired triglyceride and glucose metabolism, insulin resistance and pancreatic beta-cell hyperplasia. In this inducible model, however, this metabolic syndrome spontaneously reverses over a few weeks, in large part due to increased preadipocyte proliferation and differentiation of these preadipocytes into new adipocytes (this study and [41]). This preadipocyte proliferation and adipose tissue regeneration is not reversed by blocking the hyperglycemia associated with the lipodystrophy either by administration of an SGLT2 inhibitor (this study) or administration of leptin [41]. On other hand, this cell proliferation can be reproduced *in vitro* when primary preadipocytes are exposed to serum derived from Ai-DKO mice or conditioned media from liver from the same mice incubated *ex vivo*, but not by conditioned media from IR/IGF1R DKO adipocytes, suggesting a hepatic origin for the adipose tissue regeneration factors. This activity is also lost in protein-depleted serum after filtration with a cutoff of 3 kDa, suggesting the active molecule is likely a protein.

Analysis of the serum proteome revealed that many proteins are differently regulated in the circulation of Ai-DKO mice compared to WT, with the most upregulated being APOC3. Importantly, *in vitro* incubation with purified lipid-free human APOC3 is sufficient to induce proliferation of preadipocytes, indicating that this apolipoprotein can serve as an adipocyte growth factor. However, knockdown of APOC3 *in vivo* is not sufficient to block the proliferation of preadipocytes, indicating that APOC3 is not the only preadipocyte growth factor in the Ai-DKO mice.

Adipogenesis is complex and occurs via three major cellular processes: (1) commitment of multipotent mesenchymal cells to a preadipocyte lineage; (2) preadipocyte proliferation and (3) adipocyte differentiation [11]. Each of these steps can be regulated by intrinsic and extrinsic factors. For instance, precursors isolated from different adipose depots exhibit different proliferative and differentiation capacity [22,26,42]. Similarly, environmental cues such as nutrient availability, sex hormones or disease state also affects adipogenesis [17,53]. In Ai-DKO mouse, we find that both white and brown adipocyte regenerate over time, but recovery of brown fat takes longer, both in terms of recovery of its mass and its thermogenic function [41]. Morphologically, the regenerated brown adipocytes exhibit both unilocular and multilocular lipid droplets, more closely resembling human BAT than typical intrascapular murine BAT. What causes this heterogeneity is unclear, but Song et al. have recently shown the presence of two subtypes of adipocytes within BAT: one highly responsive to adrenergic activation and heat production, the other with relatively lower thermogenic capacity and larger lipid droplets [47]. In addition, each BAT adipocyte subtype is modulated by different metabolic stressors, including hypoxia and aging. Likewise, different subtypes of white preadipocytes have been identified [22]. Thus, it is possible that the metabolic and structural changes that occurred during BAT and WAT regeneration in Ai-DKO mice favor the differentiation of different subtypes, including the more lipogenic subtype of brown adipocyte.

Another potential factor driving adipogenesis in Ai-DKO mice is hyperinsulinemia. Exposure to high insulin levels combined with excess of energy substrate is known to modulate adipogenesis via both increases in adipocyte size (hypertrophy) and increases in adipocyte number (hyperplasia) [13,18,19,51,53]. Using leptin treatment [41] or treatment with an SGLT2 inhibitor *in vivo* (this study), we show that preadipocyte proliferation occurs independent of changes in circulating insulin, glucose, or triglycerides levels. More importantly, preadipocyte proliferation can be reproduced *in vitro* when normal cells are incubated with serum derived from Ai-DKO mice, but this is blunted when proteins are depleted from the serum by filtration with a 3 kDa membrane, suggesting the presence of a circulating protein or proteins that drive preadipocyte proliferation. Interestingly, although Ai-DKO mice also exhibit beta-cell proliferation, serum from Ai-DKO does not stimulate the proliferation of MIN6 cells *in vitro* suggesting that different growth factors [20,34] are likely involved in regulation of beta-cell and preadipocyte proliferation, although other requirements may also be different.

Adipocytes are known to secrete a wide variety of molecules, including multiple proteins [10,48], lipid species [23,57] and exosomal miRNAs [35,50]. These help to regulate the metabolism and function of neighboring cells, as well as that of distal organs. In the adipose tissue depot itself, this communication between cells can involve mature adipocytes, endothelial cells, tissue resident inflammatory cells/macrophages and preadipocytes. This communication is fundamental to adipose tissue development and function [2,6,16,29,56]. In this study, however, we find that preadipocyte proliferation is not induced using conditioned media from i-DKO adipose cells themselves, suggesting that i-DKO adipocytes are not the source of the growth factor.

To identify the adipotrophic factors involved in tissue crosstalk, we therefore performed an unbiased proteomic analysis of Ai-DKO mice serum. This revealed 107 proteins differentially regulated by at least 1.25-fold in Ai-DKO serum compared to WT. As expected, adipokines, such as adipsin and adiponectin are significant lower in Ai-DKO serum compared to WT serum at Day 3. Interestingly, their levels are not completely reversed over the period of 30 days despite return of the majority of the fat mass, suggesting that even after regeneration, the secretory capacity of these new adipocytes is still impaired. Additionally, we observe that this adipose tissue remodeling is associated with increased levels of circulating collagens. Whether collagens are being secreted by the Ai-DKO adipocytes, the proliferating preadipocytes, or other cell types, such as liver, is not clear. However, collagens are important to adipose tissue function, playing a major role in extracellular matrix (ECM) remodeling, which in turn can facilitate cellular adaptation to nutrient availability, including changes in cell size and number [28,40]. It is also possible that the circulating collagens are derived from the ECM surrounding apoptotic adipocytes, and their degradation facilitates preadipocyte proliferation.

Among the 47 differentially regulated proteins in the serum of Ai-DKO mice at Day 30, we find lower levels of creatine kinase M and B (KCRM and KCRB). Reduced levels of this kinase, specially the skeletal muscle isoform (KCRM), are associated with reduced physical activity, muscle loss and muscle atrophy [38]. Lipodystrophy due to deletion of IR and IGF1R receptors in adipose tissue, however, does not reduce spontaneous activity [3] or result in loss of skeletal muscle weight (*data not shown*), suggesting that low KCRM levels in serum of Ai-DKO mice are not driven by these factors. Glutathione S-transferase omega-1 (GST01) is a gene normally highly expressed in the liver and immune cells such as macrophages where it functions as a deglutathionylating enzyme thereby promoting inflammasome activation and secretion of interleukin-1β (IL-1β) [15]. Whole body deletion of GSTO1 increases resistance to inflammatory lipopolysaccharides and diet-induced obesity [30]. Thus, lower levels of GSTO1 in the circulation of Ai-DKO mice suggests that their capacity to cope with diet-induced inflammation or bacterial infection might be altered, although this was not examined.

Thirty-two proteins were upregulated in Ai-DKO serum at Day 30 including complement factors 6 and 8 and EGFR. Increased levels of complement proteins have been previously observed in obesity, insulin resistance, and type 2 diabetes mellitus [[9], [36], [37], [45]]. Similarly, increased level of circulating soluble EGFR have been shown to positively correlate with hepatic insulin resistance in both obese mice and people with type 2 diabetes [21]. The abundance of these proteins also correlated with the insulin resistance present in the Ai-DKO mice at Day 30 [41], suggesting a positive correlation between the levels of these proteins and the impairment in whole-body metabolism. Whether elevated C6, C8 and EGFR are the cause or consequence of insulin resistance still needs to be determined.

Many apolipoproteins are also elevated in serum of Ai-DKO mice, including APOC3, which was the most upregulated apolipoprotein, exhibiting over 2.5- and 1.9-fold increases at Days 3 and 30, respectively. Apolipoproteins are structural components of circulating lipoproteins. These lipoproteins control flux and metabolism of many lipids and cholesterol. Apolipoproteins are mainly synthesized and secreted by the hepatocytes and enterocytes [27]. APOC3, for instance, is present in chylomicrons, very low-density (VLDL), low-density (LDL) and high-density (HDL) lipoproteins [33,55]. At the cellular level, APOC3 is known to interact with the scavenger receptor, class B type 1 (SR-B1), which is important for cholesterol efflux to HDL [7,55]. It also delays clearance of triglyceride-rich lipoproteins by hepatocytes [12]. APOC3 can activate the inflammasome pathway in monocytes via binding to toll-like receptors 2 and 4 [58] and can induce inflammation in endothelial cells [49]. Additionally, relevant to the current study, APOC3-enriched VLDL has been shown to induce smooth muscle cell proliferation via activation of the AKT signaling pathway [24]. This is similar to our finding that preadipocyte growth is stimulated when cells are incubated with conditioned media from liver from Ai-DKO mice *ex vivo.* This effect is blocked when the media is treated with antibody against APOC3, and this is mimicked when preadipocytes cells are treated with purified lipid-free human APOC3.

To determine if APOC3 was the only factor contributing to adipose tissue regeneration *in vivo*, we knocked-down APOC3 using liver-targeting siRNAs. We observed that lowering APOC3 in Ai-DKO mice leads to downregulation of the expression of lipogenic genes in the liver and partially reverses the hypertriglyceridemia, but it does not affect hyperglycemia or hyperinsulinemia. Likewise, reducing APOC3 levels does not reverse the increase in preadipocyte proliferation which occurs *in vivo*, suggesting that while APOC3 may contribute to the preadipocyte proliferation observed in Ai-DKO mice, it is not the only circulating growth factor controlling adipocyte remodeling. While our data suggest that Ai-DKO adipocytes do not themselves secrete an adipocyte growth factor, we don't exclude the possibility that other cells within the fat depot, including endothelial, smooth muscle or immune cells, may secrete molecules that could communicate with preadipocytes and, in collaboration with APOC3, induce their proliferation. It is also possible that loss of adipocytes induced by the deletion of IR/IGF1R creates a mechanical stress in the fat pad that could signal to one of the adipose tissue resident cells leading to preadipocyte proliferation. Whether these mechanisms also contribute to the adipose tissue regeneration in Ai-DKO mice need to be determined.

In summary, loss of IR/IGF1R receptors in mature adipocytes leads to a severe lipodystrophy phenotype marked by lipid accumulation in non-adipose organs and multiple metabolic derangements. However, when induced by transient activation of the Cre recombinase, these metabolic changes are reversed within weeks, at least in part due to preadipocyte proliferation and regeneration of adipose tissue. The balance between adipose tissue loss and regeneration appears to be largely controlled by circulating factors derived from liver. One of these hepatocyte derived factors is apolipoprotein C3, which is elevated in Ai-DKO mice and is sufficient to induce preadipocyte proliferation *in vitro.* However*,* knockdown of APOC3 is not sufficient to block adipose tissue regeneration *in vivo*. Thus, maintenance of adipose tissue mass represents a homeostatic program which is controlled, at least in part, by circulating molecules such as APOC3 and others whose nature of which remains to be determined. Identification of these adipocyte growth factors could lead to new approaches to the treatment of both obesity and some forms of lipodystrophy in humans.

## 4 Experimental procedures

All protocols were approved by the Institutional Animal Care and Use Committee of the Joslin Diabetes Center and the University of Massachusetts Medical School and were in accordance with National Institutes of Health guidelines.

### 4.1 Animal studies

Male mice were used for all studies unless otherwise indicated. Adiponectin-CreERT2 mice were a generous gift from the Stefan Offermans (Max-Planck-Institute for Heart and Lung Research, Bad Nauheim, German) and can now be purchased at Jackson Laboratories (stock no. 025124). IR and IGF1R floxed mice were created in this lab as previously described [3]. Control and fat-specific inducible double KO (Ai-DKO) mice were maintained on a C57Bl/6 background by breeding Adiponectin-CreERT2 IR f/f and IGF1R f/f with IR f/f and IGF1Rf/f mice. Additional information regarding the animal studies is in Appendix A.

### 4.2 Glucose and Insulin tolerance tests

Glucose and Insulin tolerance tests were performed as described in Appendix A.

### 4.3 Tissue and serum analysis

Tissue weight and serum protein and metabolites were measured as described in Appendix A.

### 4.4 Beta-cell histology, proliferation, and islet area

Methods for analysis of beta-cell histology, immunofluorescence and gene expression for proliferation markers are in Appendix A.

### 4.5 Serum proteomics using LC-MS/MS

80 μL of serum samples from the WT Day 3, Ai-DKO Day 3, and A-iDKO Day 30 conditions were subjected proteomics using LC-MS/MS as detailed in Appendix A.

### 4.6 Generation of i-DKO cells and adipocyte differentiation

Isolation, immortalization, differentiation, and tamoxifen-induced deletion of IR/IGFR in i-DKO cells were performed as described in Appendix A.

### 4.7 Thymidine incorporation

Thymidine incorporation in preadipocyte-enriched stromal vascular fraction (SFV) and in MIN6 cell line was performed as described in Appendix A*.*

### 4.8 Ex vivo liver culture

On Day 3 after the last tamoxifen injection, WT and Ai-DKO mice were sacrificed. A liver slice (∼100 mg) was dissected, washed twice with PBS, and then cultured in 1 ml of serum-free Dulbecco's modified Eagle's medium (DMEM) with 5.5 mM glucose for 6 h at 37 °C, after which, the conditioned media was collected, centrifuged for 10 min at 12,000 g, and kept at −80 °C.

### 4.9 Sorting of adipocyte precursor

Isolated SVF was resuspended in cold Hank's balanced salt solution (HBSS) with 2% fetal bovine serum (FBS) and cell sorting was performed as described in Appendix A.

### 4.10 Protein extraction and immunoblot analysis

Tissues were homogenized and western blot was performed as described in Appendix A*.*

### 4.11 RNA extraction and gene expression

RNA was extracted using TRIZOL as described in Appendix A.

### 4.12 Statistical analyses

Data are presented as mean ± SEM. Comparisons between two groups were analyzed using an unpaired two-tailed Student t test. Comparison between more than two groups was performed using one-way ANOVA or two-way ANOVA with repeated measures followed by post hoc t tests, as appropriate. Statistical analysis was performed using Graph Pad Prism (Version 9.02). Significance level was set at ∗p < 0.05, ∗∗p < 0.01, ∗∗∗p < 0.001, and ∗∗∗∗p < 0.0001. *Moderated F-tests and hierarchical clustering:* To discover the differential proteins, the R package limma that powers differential expression analyses was used (Ritchie et al., 2015). A moderated F-test was performed to detect proteins that were differentially expressed among the 3 different groups, namely Ctrl Day 3, Ai-DKO Day 3, and Ai-DKO Day 30. Proteins that had FDR <0.05 and |FC| > 1.25 in the F-tests were selected (total of 107). Proteins were clustered in a hierarchical dendrogram using a variable cut height approach (Langfelder, Zhang, and Horvath 2008). Four clusters were identified according to the hierarchical tree.

## Conflict of interest

None declared.

## Data Availability

Data will be made available on request.
