# Supplementary material for: Apolipoprotein C3 and circulating mediators of preadipocyte proliferation in states of lipodystrophy
Source: Mol Metab. 2022 Aug 11;64:101572. doi: 10.1016/j.molmet.2022.101572 (PMC9418991; doi:10.1016/j.molmet.2022.101572)
Supplement: Multimedia component 1 [file mmc1.pptx]

## Slide 1
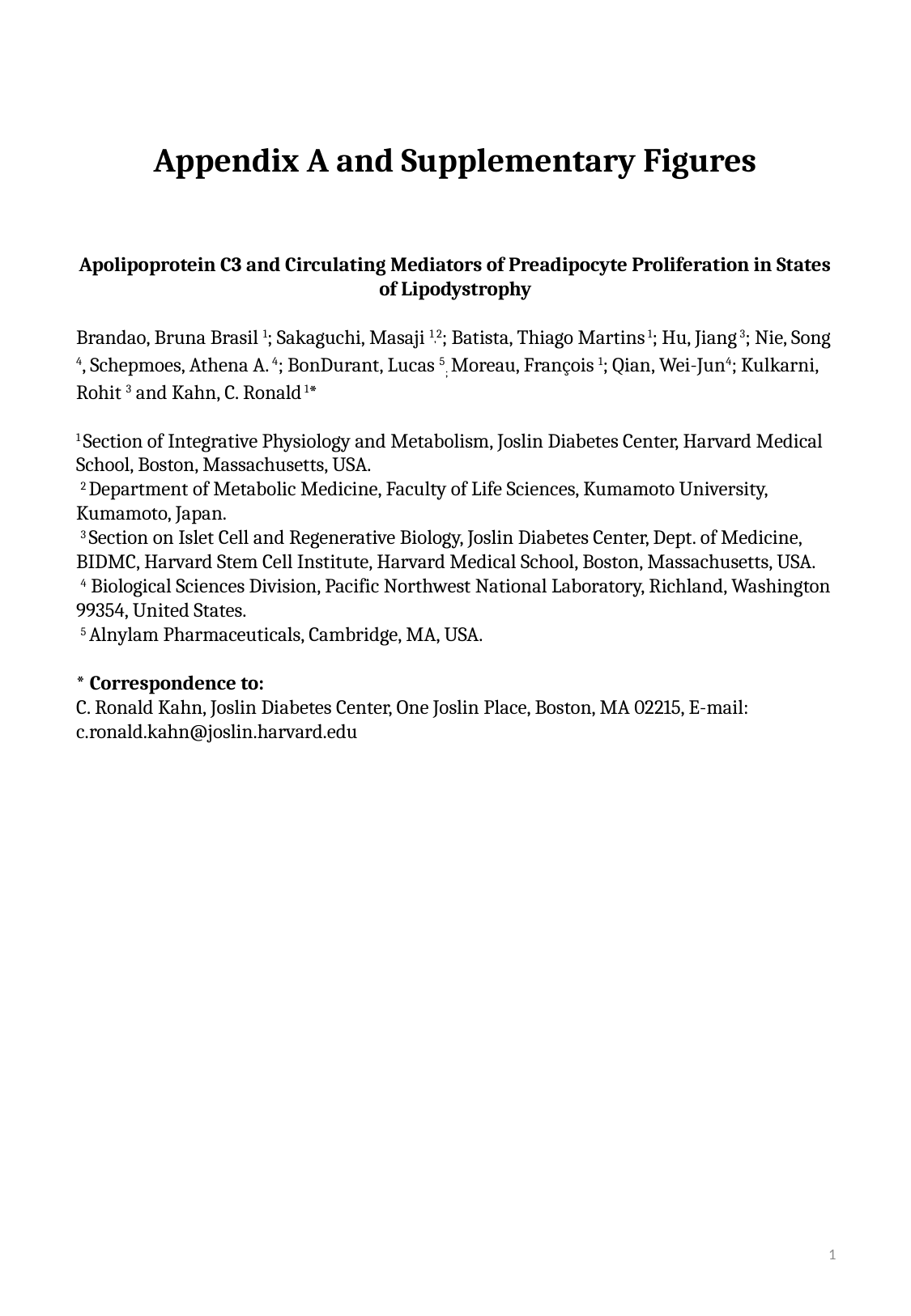

Appendix A and Supplementary Figures
Apolipoprotein C3 and Circulating Mediators of Preadipocyte Proliferation in States of Lipodystrophy
Brandao, Bruna Brasil 1; Sakaguchi, Masaji 1’2; Batista, Thiago Martins 1; Hu, Jiang 3; Nie, Song 4, Schepmoes, Athena A. 4; BonDurant, Lucas 5; Moreau, François 1; Qian, Wei-Jun4; Kulkarni, Rohit 3 and Kahn, C. Ronald 1*
1 Section of Integrative Physiology and Metabolism, Joslin Diabetes Center, Harvard Medical School, Boston, Massachusetts, USA.
 2 Department of Metabolic Medicine, Faculty of Life Sciences, Kumamoto University, Kumamoto, Japan.
 3 Section on Islet Cell and Regenerative Biology, Joslin Diabetes Center, Dept. of Medicine, BIDMC, Harvard Stem Cell Institute, Harvard Medical School, Boston, Massachusetts, USA.
 4 Biological Sciences Division, Pacific Northwest National Laboratory, Richland, Washington 99354, United States.
 5 Alnylam Pharmaceuticals, Cambridge, MA, USA.
* Correspondence to:
C. Ronald Kahn, Joslin Diabetes Center, One Joslin Place, Boston, MA 02215, E-mail: c.ronald.kahn@joslin.harvard.edu
1

## Slide 2
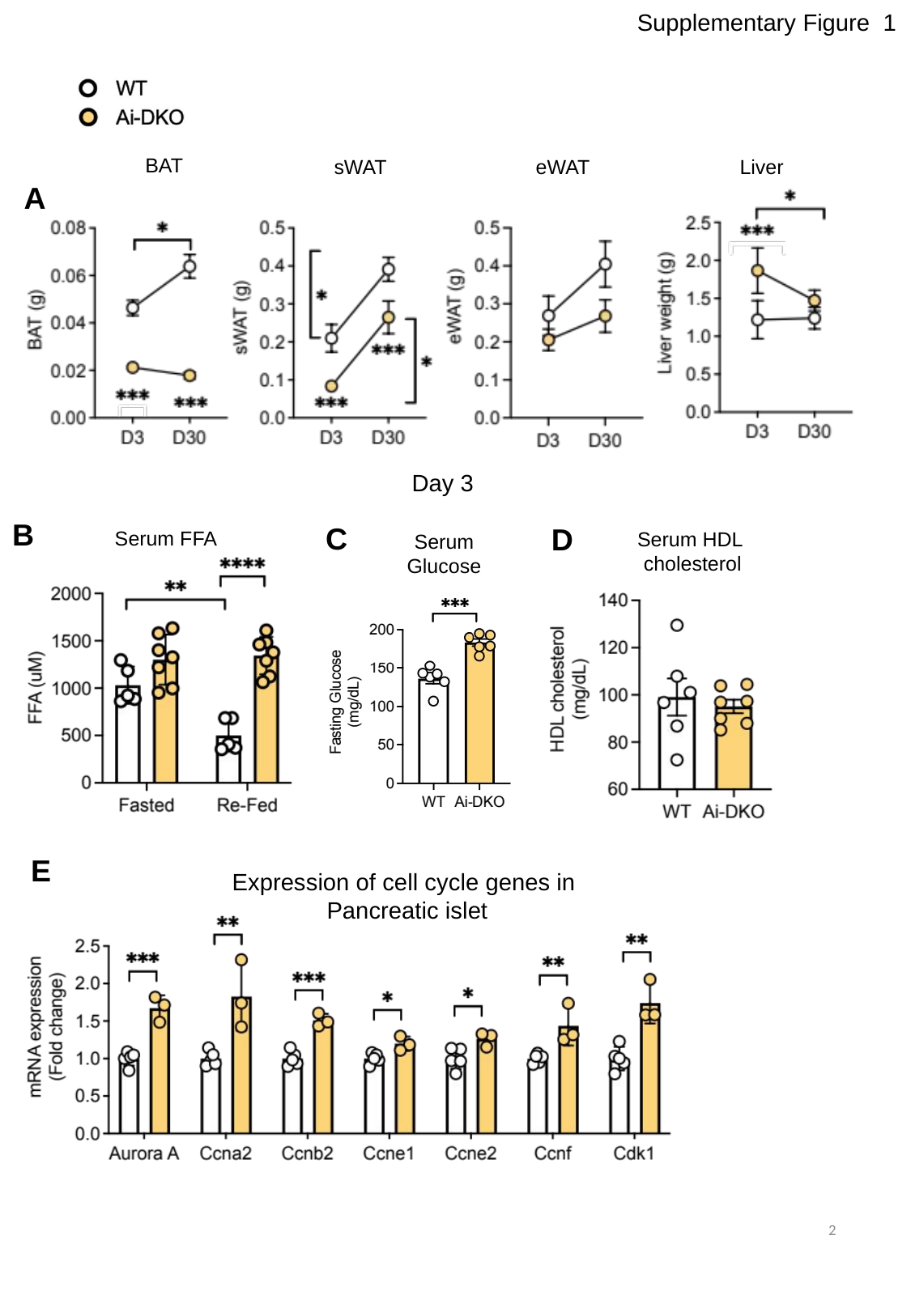

Supplementary Figure 1
BAT
eWAT
sWAT
Liver
A
Day 3
B
C
D
Serum FFA
Serum HDL
cholesterol
Serum
Glucose
E
Expression of cell cycle genes in
 Pancreatic islet
2

## Slide 3
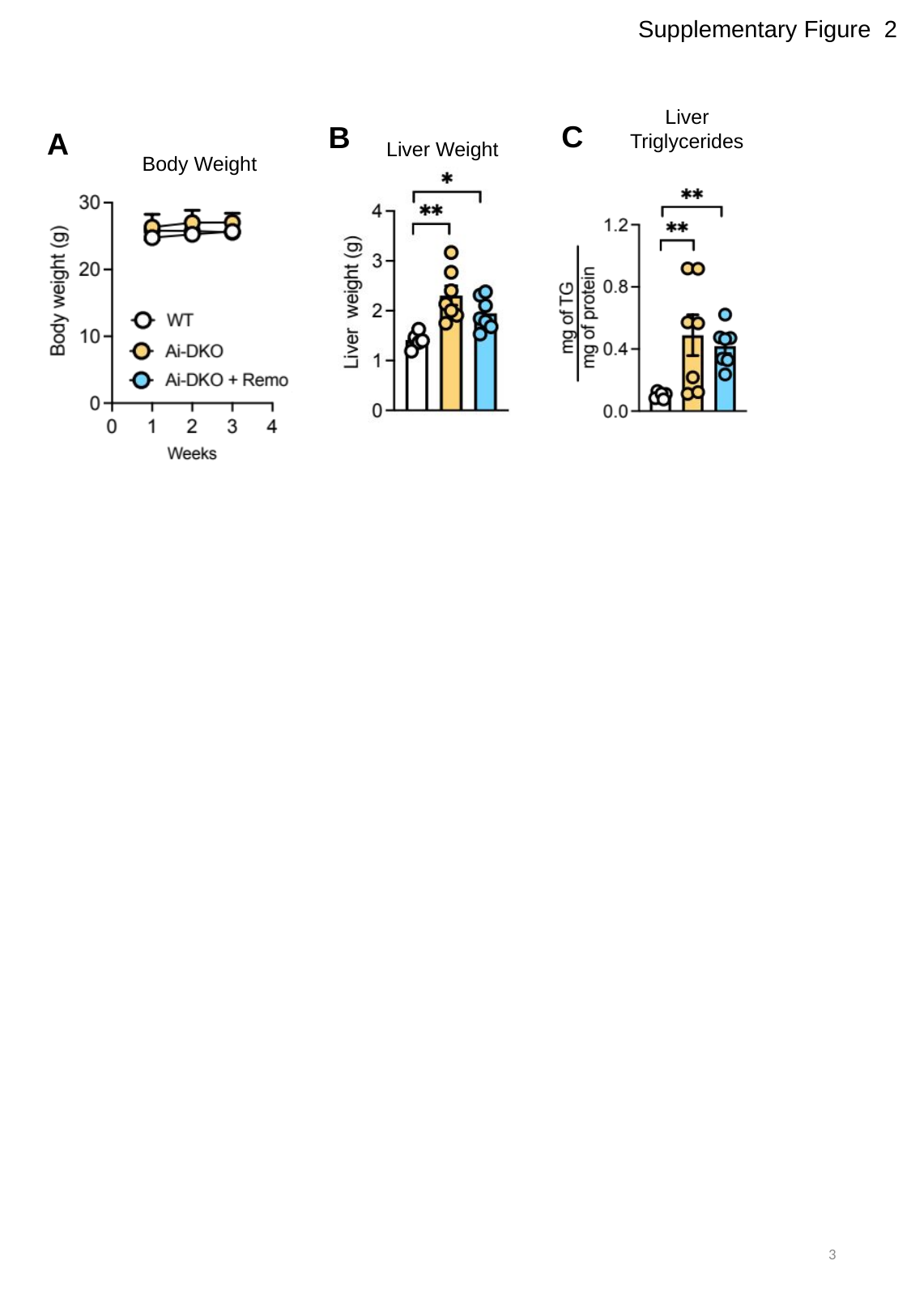

Supplementary Figure 2
Liver
Triglycerides
C
B
A
Liver Weight
Body Weight
3

## Slide 4
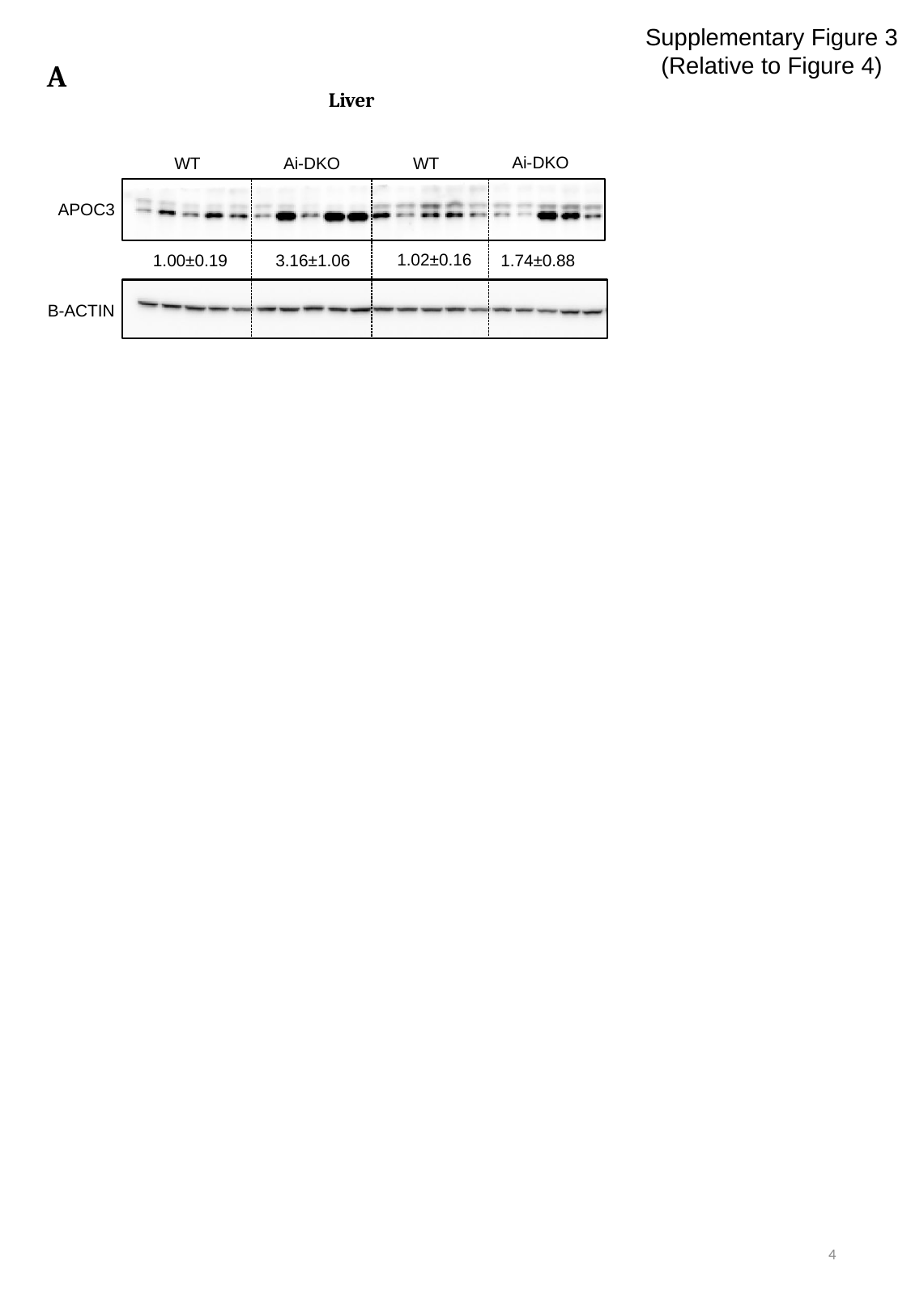

Supplementary Figure 3
(Relative to Figure 4)
A
Liver
Ai-DKO
WT
Ai-DKO
WT
APOC3
1.02±0.16
1.74±0.88
1.00±0.19
3.16±1.06
B-ACTIN
4

## Slide 5
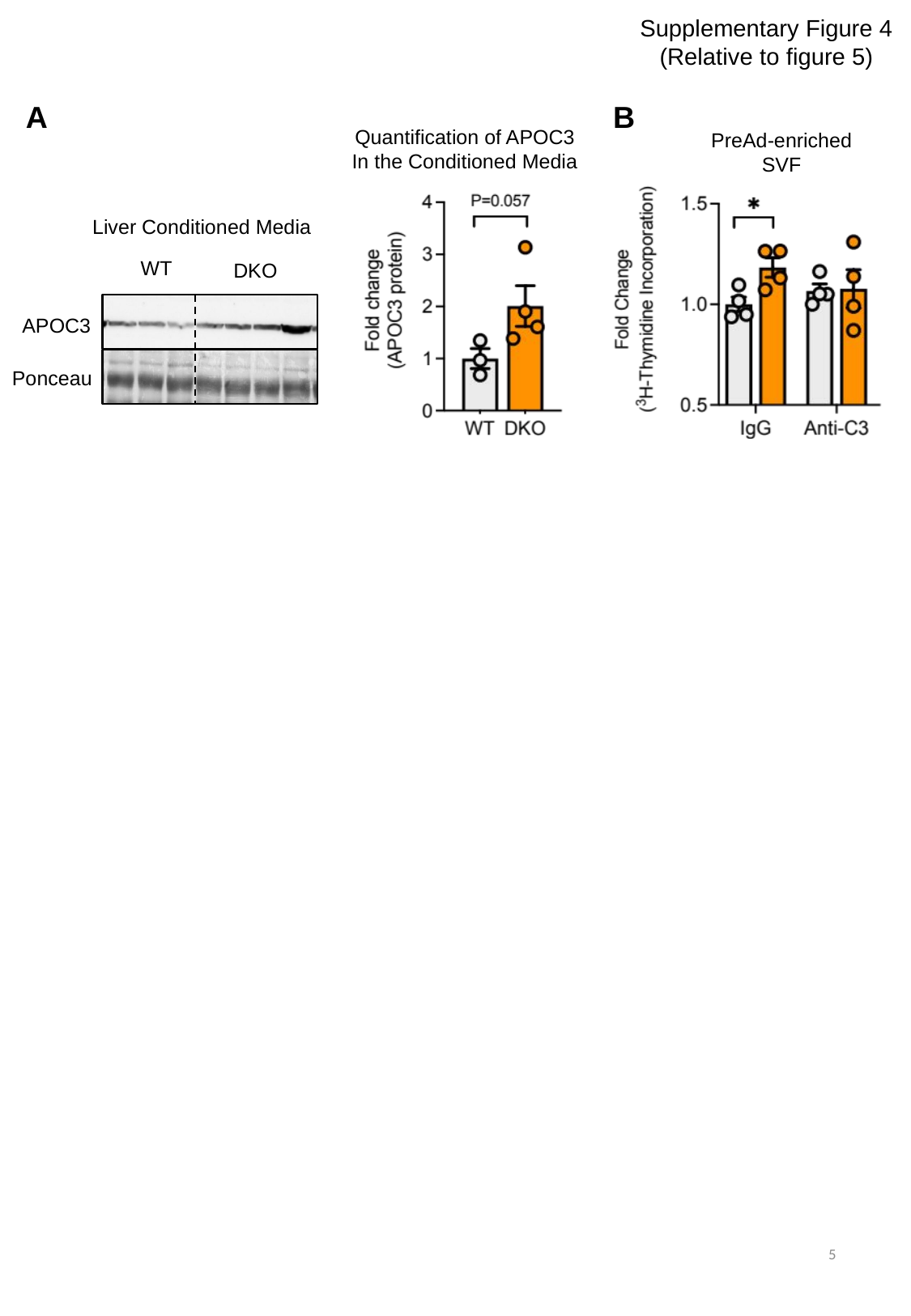

Supplementary Figure 4
(Relative to figure 5)
A
B
Quantification of APOC3
In the Conditioned Media
PreAd-enriched
SVF
Liver Conditioned Media
WT
DKO
APOC3
Ponceau
5

## Slide 6
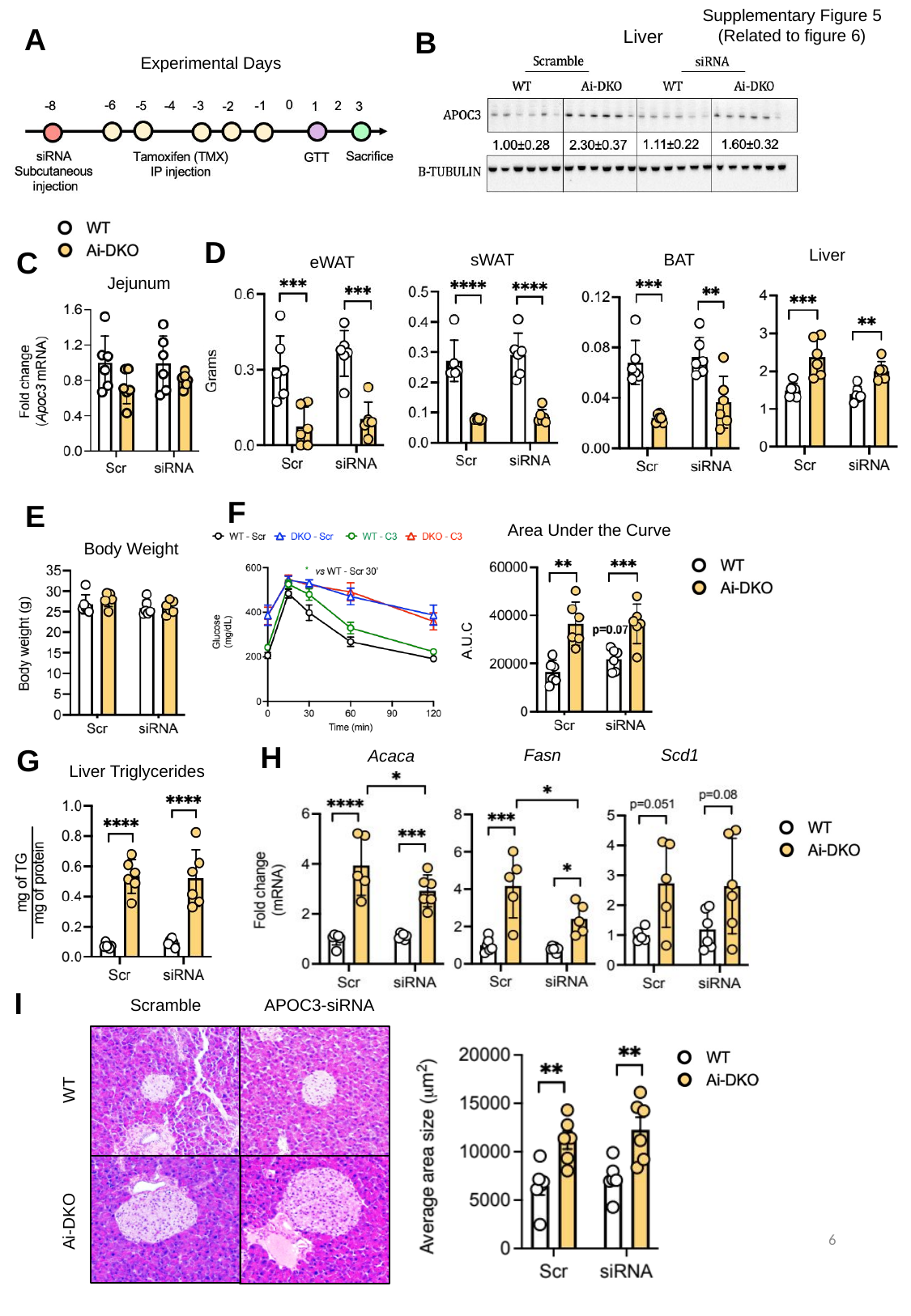

Supplementary Figure 5
(Related to figure 6)
A
B
Liver
Experimental Days
D
Liver
sWAT
BAT
eWAT
C
Jejunum
F
E
Area Under the Curve
Body Weight
H
G
Scd1
Fasn
Acaca
Liver Triglycerides
I
Scramble
APOC3-siRNA
WT
6
Ai-DKO

## Slide 7
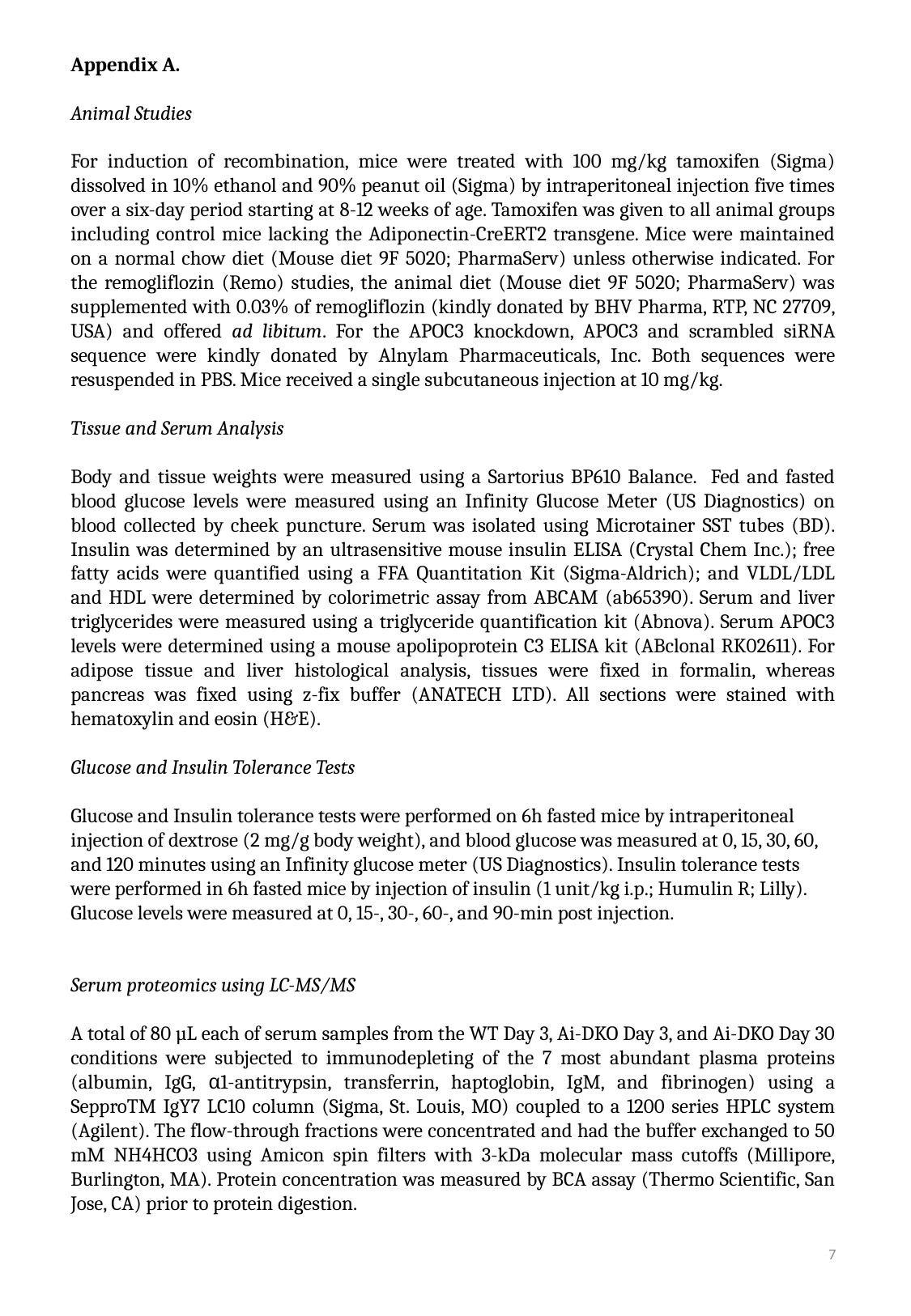

Appendix A.
Animal Studies
For induction of recombination, mice were treated with 100 mg/kg tamoxifen (Sigma) dissolved in 10% ethanol and 90% peanut oil (Sigma) by intraperitoneal injection five times over a six-day period starting at 8-12 weeks of age. Tamoxifen was given to all animal groups including control mice lacking the Adiponectin-CreERT2 transgene. Mice were maintained on a normal chow diet (Mouse diet 9F 5020; PharmaServ) unless otherwise indicated. For the remogliflozin (Remo) studies, the animal diet (Mouse diet 9F 5020; PharmaServ) was supplemented with 0.03% of remogliflozin (kindly donated by BHV Pharma, RTP, NC 27709, USA) and offered ad libitum. For the APOC3 knockdown, APOC3 and scrambled siRNA sequence were kindly donated by Alnylam Pharmaceuticals, Inc. Both sequences were resuspended in PBS. Mice received a single subcutaneous injection at 10 mg/kg.
Tissue and Serum Analysis
Body and tissue weights were measured using a Sartorius BP610 Balance. Fed and fasted blood glucose levels were measured using an Infinity Glucose Meter (US Diagnostics) on blood collected by cheek puncture. Serum was isolated using Microtainer SST tubes (BD). Insulin was determined by an ultrasensitive mouse insulin ELISA (Crystal Chem Inc.); free fatty acids were quantified using a FFA Quantitation Kit (Sigma-Aldrich); and VLDL/LDL and HDL were determined by colorimetric assay from ABCAM (ab65390). Serum and liver triglycerides were measured using a triglyceride quantification kit (Abnova). Serum APOC3 levels were determined using a mouse apolipoprotein C3 ELISA kit (ABclonal RK02611). For adipose tissue and liver histological analysis, tissues were fixed in formalin, whereas pancreas was fixed using z-fix buffer (ANATECH LTD). All sections were stained with hematoxylin and eosin (H&E).
Glucose and Insulin Tolerance Tests
Glucose and Insulin tolerance tests were performed on 6h fasted mice by intraperitoneal injection of dextrose (2 mg/g body weight), and blood glucose was measured at 0, 15, 30, 60, and 120 minutes using an Infinity glucose meter (US Diagnostics). Insulin tolerance tests were performed in 6h fasted mice by injection of insulin (1 unit/kg i.p.; Humulin R; Lilly). Glucose levels were measured at 0, 15-, 30-, 60-, and 90-min post injection.
Serum proteomics using LC-MS/MS
A total of 80 µL each of serum samples from the WT Day 3, Ai-DKO Day 3, and Ai-DKO Day 30 conditions were subjected to immunodepleting of the 7 most abundant plasma proteins (albumin, IgG, α1-antitrypsin, transferrin, haptoglobin, IgM, and fibrinogen) using a SepproTM IgY7 LC10 column (Sigma, St. Louis, MO) coupled to a 1200 series HPLC system (Agilent). The flow-through fractions were concentrated and had the buffer exchanged to 50 mM NH4HCO3 using Amicon spin filters with 3-kDa molecular mass cutoffs (Millipore, Burlington, MA). Protein concentration was measured by BCA assay (Thermo Scientific, San Jose, CA) prior to protein digestion.
7

## Slide 8
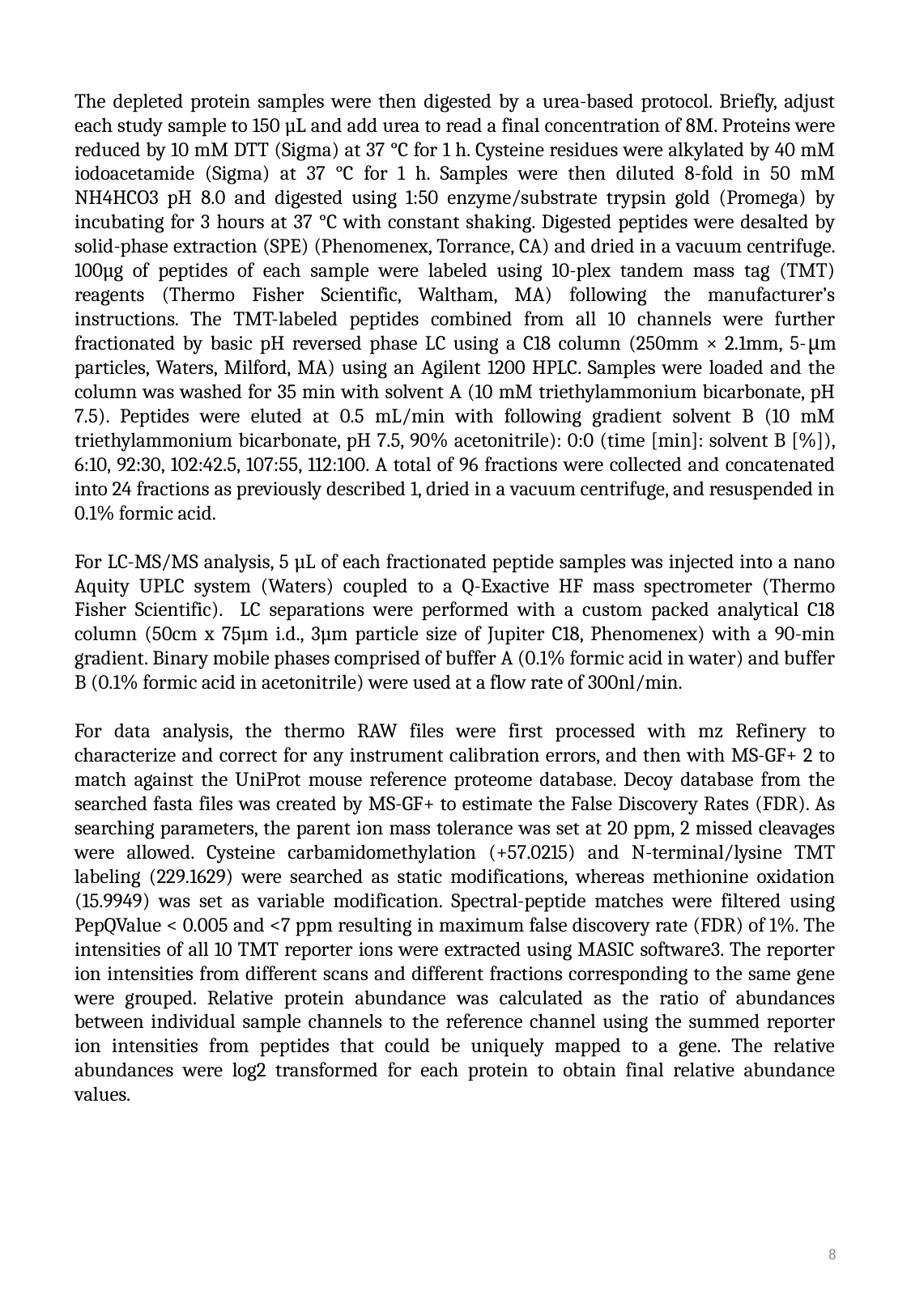

The depleted protein samples were then digested by a urea-based protocol. Briefly, adjust each study sample to 150 µL and add urea to read a final concentration of 8M. Proteins were reduced by 10 mM DTT (Sigma) at 37 °C for 1 h. Cysteine residues were alkylated by 40 mM iodoacetamide (Sigma) at 37 °C for 1 h. Samples were then diluted 8-fold in 50 mM NH4HCO3 pH 8.0 and digested using 1:50 enzyme/substrate trypsin gold (Promega) by incubating for 3 hours at 37 °C with constant shaking. Digested peptides were desalted by solid-phase extraction (SPE) (Phenomenex, Torrance, CA) and dried in a vacuum centrifuge. 100µg of peptides of each sample were labeled using 10-plex tandem mass tag (TMT) reagents (Thermo Fisher Scientific, Waltham, MA) following the manufacturer’s instructions. The TMT-labeled peptides combined from all 10 channels were further fractionated by basic pH reversed phase LC using a C18 column (250mm × 2.1mm, 5-μm particles, Waters, Milford, MA) using an Agilent 1200 HPLC. Samples were loaded and the column was washed for 35 min with solvent A (10 mM triethylammonium bicarbonate, pH 7.5). Peptides were eluted at 0.5 mL/min with following gradient solvent B (10 mM triethylammonium bicarbonate, pH 7.5, 90% acetonitrile): 0:0 (time [min]: solvent B [%]), 6:10, 92:30, 102:42.5, 107:55, 112:100. A total of 96 fractions were collected and concatenated into 24 fractions as previously described 1, dried in a vacuum centrifuge, and resuspended in 0.1% formic acid.
For LC-MS/MS analysis, 5 µL of each fractionated peptide samples was injected into a nano Aquity UPLC system (Waters) coupled to a Q-Exactive HF mass spectrometer (Thermo Fisher Scientific). LC separations were performed with a custom packed analytical C18 column (50cm x 75µm i.d., 3µm particle size of Jupiter C18, Phenomenex) with a 90-min gradient. Binary mobile phases comprised of buffer A (0.1% formic acid in water) and buffer B (0.1% formic acid in acetonitrile) were used at a flow rate of 300nl/min.
For data analysis, the thermo RAW files were first processed with mz Refinery to characterize and correct for any instrument calibration errors, and then with MS-GF+ 2 to match against the UniProt mouse reference proteome database. Decoy database from the searched fasta files was created by MS-GF+ to estimate the False Discovery Rates (FDR). As searching parameters, the parent ion mass tolerance was set at 20 ppm, 2 missed cleavages were allowed. Cysteine carbamidomethylation (+57.0215) and N-terminal/lysine TMT labeling (229.1629) were searched as static modifications, whereas methionine oxidation (15.9949) was set as variable modification. Spectral-peptide matches were filtered using PepQValue < 0.005 and <7 ppm resulting in maximum false discovery rate (FDR) of 1%. The intensities of all 10 TMT reporter ions were extracted using MASIC software3. The reporter ion intensities from different scans and different fractions corresponding to the same gene were grouped. Relative protein abundance was calculated as the ratio of abundances between individual sample channels to the reference channel using the summed reporter ion intensities from peptides that could be uniquely mapped to a gene. The relative abundances were log2 transformed for each protein to obtain final relative abundance values.
8

## Slide 9
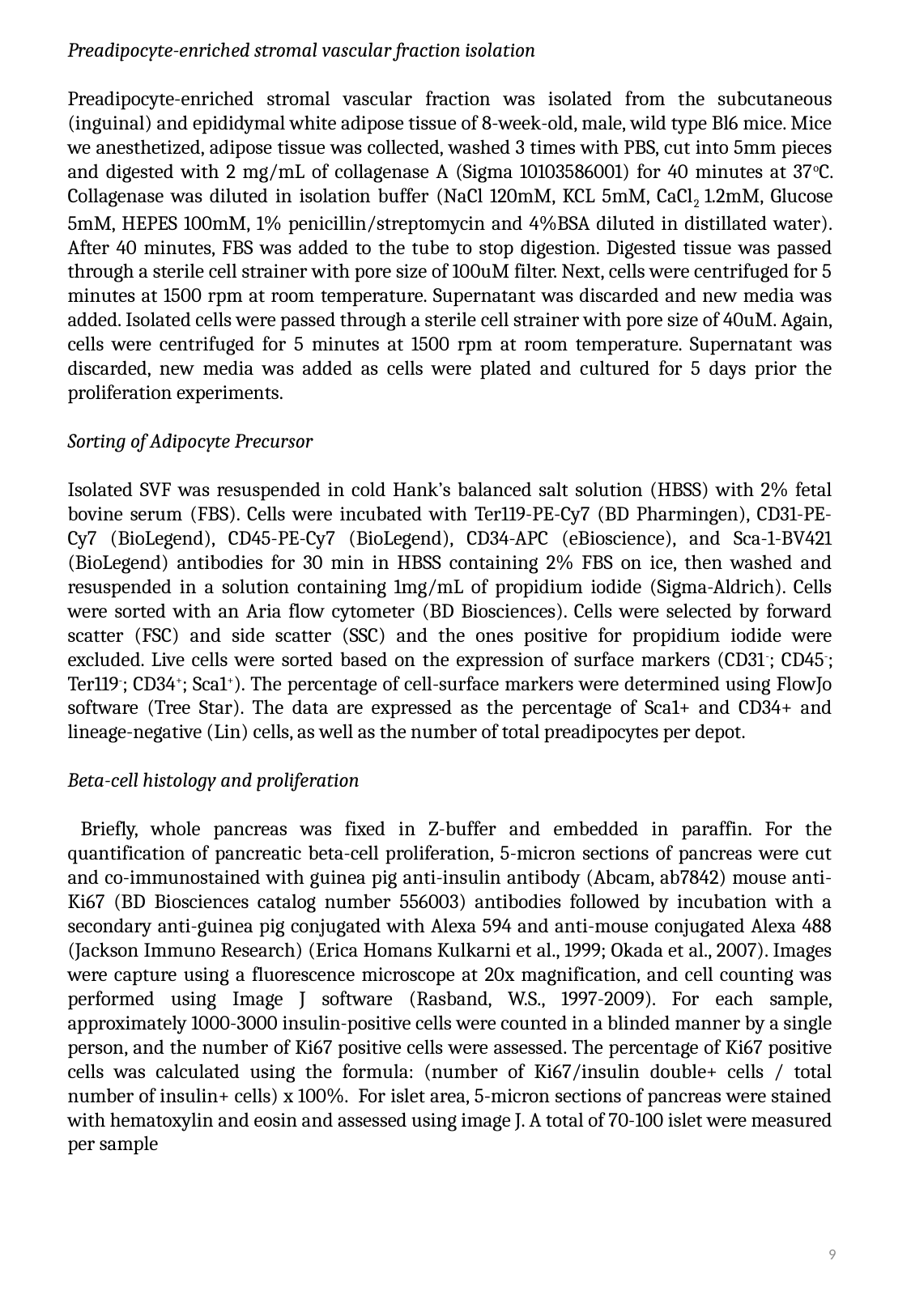

Preadipocyte-enriched stromal vascular fraction isolation
Preadipocyte-enriched stromal vascular fraction was isolated from the subcutaneous (inguinal) and epididymal white adipose tissue of 8-week-old, male, wild type Bl6 mice. Mice we anesthetized, adipose tissue was collected, washed 3 times with PBS, cut into 5mm pieces and digested with 2 mg/mL of collagenase A (Sigma 10103586001) for 40 minutes at 37oC. Collagenase was diluted in isolation buffer (NaCl 120mM, KCL 5mM, CaCl2 1.2mM, Glucose 5mM, HEPES 100mM, 1% penicillin/streptomycin and 4%BSA diluted in distillated water). After 40 minutes, FBS was added to the tube to stop digestion. Digested tissue was passed through a sterile cell strainer with pore size of 100uM filter. Next, cells were centrifuged for 5 minutes at 1500 rpm at room temperature. Supernatant was discarded and new media was added. Isolated cells were passed through a sterile cell strainer with pore size of 40uM. Again, cells were centrifuged for 5 minutes at 1500 rpm at room temperature. Supernatant was discarded, new media was added as cells were plated and cultured for 5 days prior the proliferation experiments.
Sorting of Adipocyte Precursor
Isolated SVF was resuspended in cold Hank’s balanced salt solution (HBSS) with 2% fetal bovine serum (FBS). Cells were incubated with Ter119-PE-Cy7 (BD Pharmingen), CD31-PE-Cy7 (BioLegend), CD45-PE-Cy7 (BioLegend), CD34-APC (eBioscience), and Sca-1-BV421 (BioLegend) antibodies for 30 min in HBSS containing 2% FBS on ice, then washed and resuspended in a solution containing 1mg/mL of propidium iodide (Sigma-Aldrich). Cells were sorted with an Aria flow cytometer (BD Biosciences). Cells were selected by forward scatter (FSC) and side scatter (SSC) and the ones positive for propidium iodide were excluded. Live cells were sorted based on the expression of surface markers (CD31-; CD45-; Ter119-; CD34+; Sca1+). The percentage of cell-surface markers were determined using FlowJo software (Tree Star). The data are expressed as the percentage of Sca1+ and CD34+ and lineage-negative (Lin) cells, as well as the number of total preadipocytes per depot.
Beta-cell histology and proliferation
 Briefly, whole pancreas was fixed in Z-buffer and embedded in paraffin. For the quantification of pancreatic beta-cell proliferation, 5-micron sections of pancreas were cut and co-immunostained with guinea pig anti-insulin antibody (Abcam, ab7842) mouse anti-Ki67 (BD Biosciences catalog number 556003) antibodies followed by incubation with a secondary anti-guinea pig conjugated with Alexa 594 and anti-mouse conjugated Alexa 488 (Jackson Immuno Research) (Erica Homans Kulkarni et al., 1999; Okada et al., 2007). Images were capture using a fluorescence microscope at 20x magnification, and cell counting was performed using Image J software (Rasband, W.S., 1997-2009). For each sample, approximately 1000-3000 insulin-positive cells were counted in a blinded manner by a single person, and the number of Ki67 positive cells were assessed. The percentage of Ki67 positive cells was calculated using the formula: (number of Ki67/insulin double+ cells / total number of insulin+ cells) x 100%. For islet area, 5-micron sections of pancreas were stained with hematoxylin and eosin and assessed using image J. A total of 70-100 islet were measured per sample
9

## Slide 10
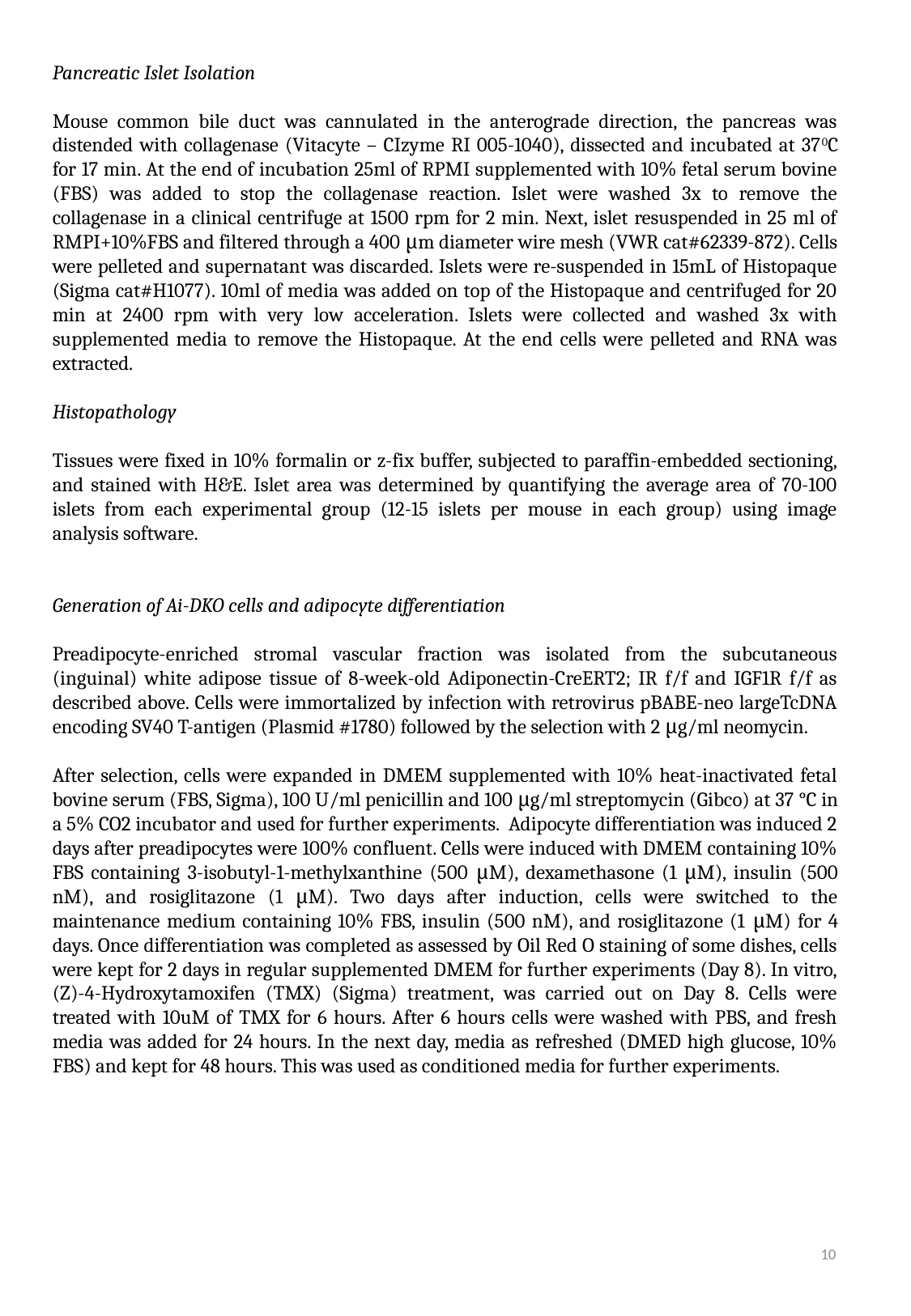

Pancreatic Islet Isolation
Mouse common bile duct was cannulated in the anterograde direction, the pancreas was distended with collagenase (Vitacyte – CIzyme RI 005-1040), dissected and incubated at 370C for 17 min. At the end of incubation 25ml of RPMI supplemented with 10% fetal serum bovine (FBS) was added to stop the collagenase reaction. Islet were washed 3x to remove the collagenase in a clinical centrifuge at 1500 rpm for 2 min. Next, islet resuspended in 25 ml of RMPI+10%FBS and filtered through a 400 μm diameter wire mesh (VWR cat#62339-872). Cells were pelleted and supernatant was discarded. Islets were re-suspended in 15mL of Histopaque (Sigma cat#H1077). 10ml of media was added on top of the Histopaque and centrifuged for 20 min at 2400 rpm with very low acceleration. Islets were collected and washed 3x with supplemented media to remove the Histopaque. At the end cells were pelleted and RNA was extracted.
Histopathology
Tissues were fixed in 10% formalin or z-fix buffer, subjected to paraffin-embedded sectioning, and stained with H&E. Islet area was determined by quantifying the average area of 70-100 islets from each experimental group (12-15 islets per mouse in each group) using image analysis software.
Generation of Ai-DKO cells and adipocyte differentiation
Preadipocyte-enriched stromal vascular fraction was isolated from the subcutaneous (inguinal) white adipose tissue of 8-week-old Adiponectin-CreERT2; IR f/f and IGF1R f/f as described above. Cells were immortalized by infection with retrovirus pBABE-neo largeTcDNA encoding SV40 T-antigen (Plasmid #1780) followed by the selection with 2 μg/ml neomycin.
After selection, cells were expanded in DMEM supplemented with 10% heat-inactivated fetal bovine serum (FBS, Sigma), 100 U/ml penicillin and 100 μg/ml streptomycin (Gibco) at 37 °C in a 5% CO2 incubator and used for further experiments. Adipocyte differentiation was induced 2 days after preadipocytes were 100% confluent. Cells were induced with DMEM containing 10% FBS containing 3-isobutyl-1-methylxanthine (500 μM), dexamethasone (1 μM), insulin (500 nM), and rosiglitazone (1 μM). Two days after induction, cells were switched to the maintenance medium containing 10% FBS, insulin (500 nM), and rosiglitazone (1 μM) for 4 days. Once differentiation was completed as assessed by Oil Red O staining of some dishes, cells were kept for 2 days in regular supplemented DMEM for further experiments (Day 8). In vitro, (Z)-4-Hydroxytamoxifen (TMX) (Sigma) treatment, was carried out on Day 8. Cells were treated with 10uM of TMX for 6 hours. After 6 hours cells were washed with PBS, and fresh media was added for 24 hours. In the next day, media as refreshed (DMED high glucose, 10% FBS) and kept for 48 hours. This was used as conditioned media for further experiments.
10

## Slide 11
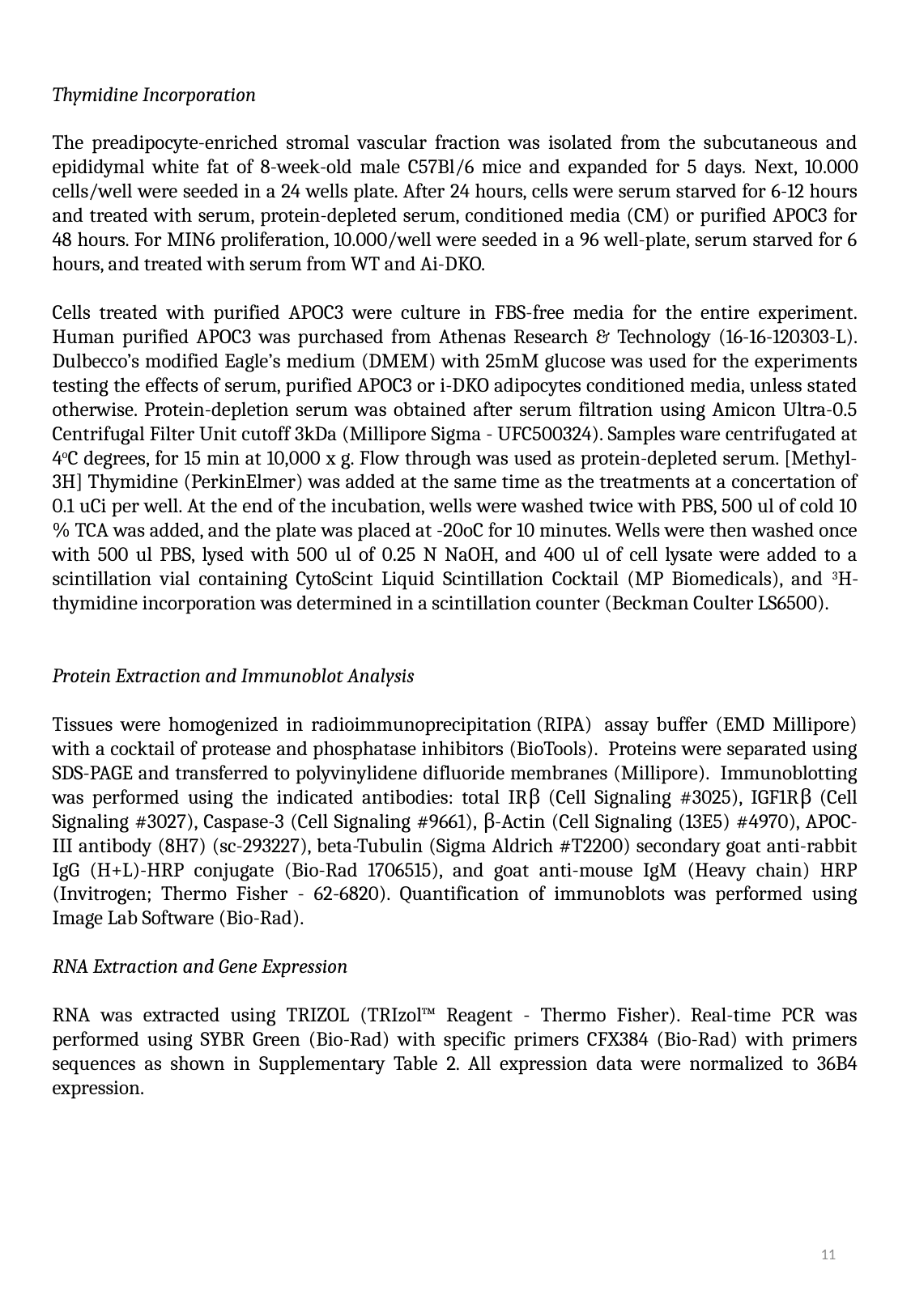

Thymidine Incorporation
The preadipocyte-enriched stromal vascular fraction was isolated from the subcutaneous and epididymal white fat of 8-week-old male C57Bl/6 mice and expanded for 5 days. Next, 10.000 cells/well were seeded in a 24 wells plate. After 24 hours, cells were serum starved for 6-12 hours and treated with serum, protein-depleted serum, conditioned media (CM) or purified APOC3 for 48 hours. For MIN6 proliferation, 10.000/well were seeded in a 96 well-plate, serum starved for 6 hours, and treated with serum from WT and Ai-DKO.
Cells treated with purified APOC3 were culture in FBS-free media for the entire experiment. Human purified APOC3 was purchased from Athenas Research & Technology (16-16-120303-L). Dulbecco’s modified Eagle’s medium (DMEM) with 25mM glucose was used for the experiments testing the effects of serum, purified APOC3 or i-DKO adipocytes conditioned media, unless stated otherwise. Protein-depletion serum was obtained after serum filtration using Amicon Ultra-0.5 Centrifugal Filter Unit cutoff 3kDa (Millipore Sigma - UFC500324). Samples ware centrifugated at 4oC degrees, for 15 min at 10,000 x g. Flow through was used as protein-depleted serum. [Methyl-3H] Thymidine (PerkinElmer) was added at the same time as the treatments at a concertation of 0.1 uCi per well. At the end of the incubation, wells were washed twice with PBS, 500 ul of cold 10 % TCA was added, and the plate was placed at -20oC for 10 minutes. Wells were then washed once with 500 ul PBS, lysed with 500 ul of 0.25 N NaOH, and 400 ul of cell lysate were added to a scintillation vial containing CytoScint Liquid Scintillation Cocktail (MP Biomedicals), and 3H-thymidine incorporation was determined in a scintillation counter (Beckman Coulter LS6500).
Protein Extraction and Immunoblot Analysis
Tissues were homogenized in radioimmunoprecipitation (RIPA)  assay buffer (EMD Millipore) with a cocktail of protease and phosphatase inhibitors (BioTools). Proteins were separated using SDS-PAGE and transferred to polyvinylidene difluoride membranes (Millipore). Immunoblotting was performed using the indicated antibodies: total IRβ (Cell Signaling #3025), IGF1Rβ (Cell Signaling #3027), Caspase-3 (Cell Signaling #9661), β-Actin (Cell Signaling (13E5) #4970), APOC-III antibody (8H7) (sc-293227), beta-Tubulin (Sigma Aldrich #T2200) secondary goat anti-rabbit IgG (H+L)-HRP conjugate (Bio-Rad 1706515), and goat anti-mouse IgM (Heavy chain) HRP (Invitrogen; Thermo Fisher - 62-6820). Quantification of immunoblots was performed using Image Lab Software (Bio-Rad).
RNA Extraction and Gene Expression
RNA was extracted using TRIZOL (TRIzol™ Reagent - Thermo Fisher). Real-time PCR was performed using SYBR Green (Bio-Rad) with specific primers CFX384 (Bio-Rad) with primers sequences as shown in Supplementary Table 2. All expression data were normalized to 36B4 expression.
11

## Slide 12
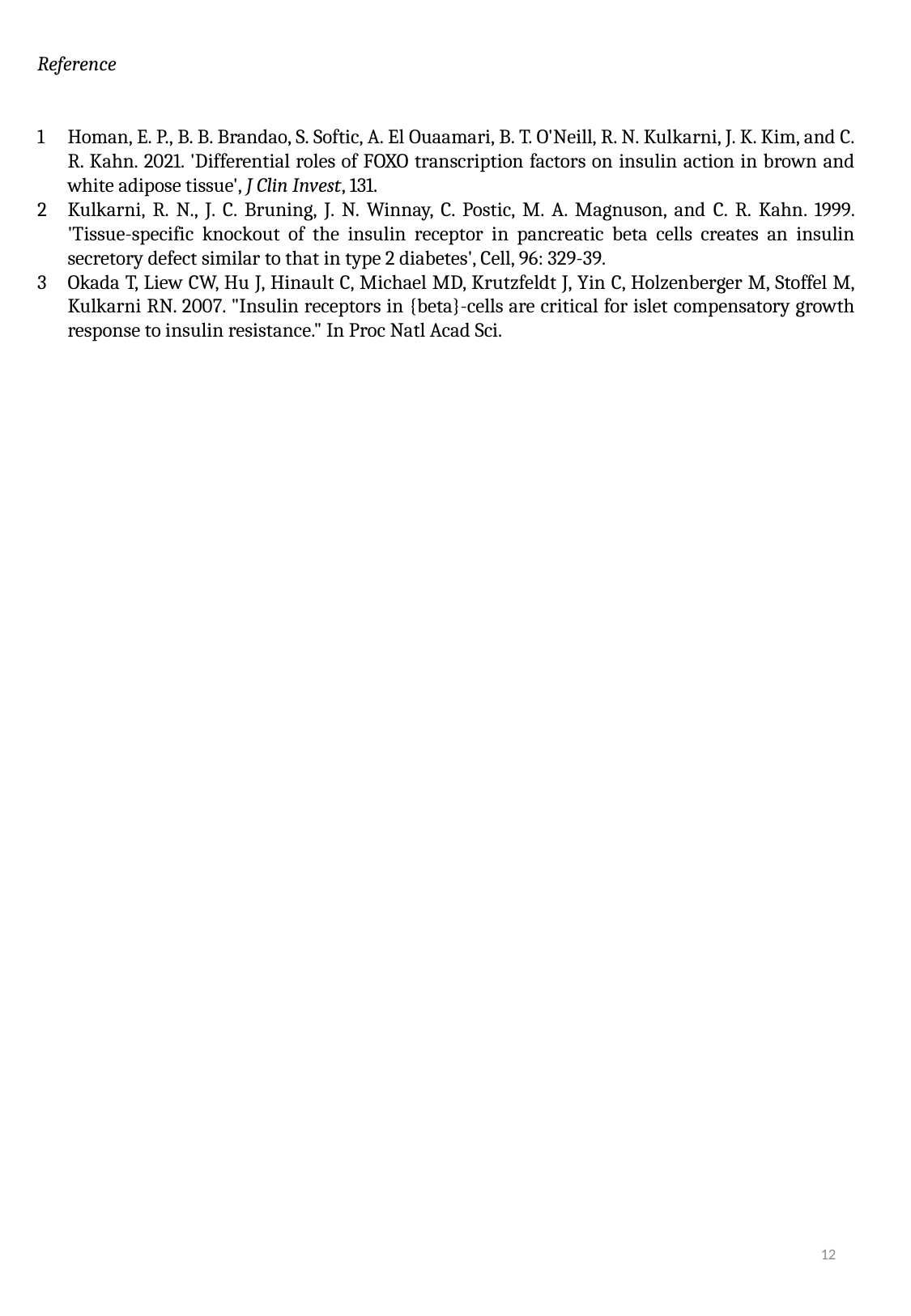

Reference
Homan, E. P., B. B. Brandao, S. Softic, A. El Ouaamari, B. T. O'Neill, R. N. Kulkarni, J. K. Kim, and C. R. Kahn. 2021. 'Differential roles of FOXO transcription factors on insulin action in brown and white adipose tissue', J Clin Invest, 131.
Kulkarni, R. N., J. C. Bruning, J. N. Winnay, C. Postic, M. A. Magnuson, and C. R. Kahn. 1999. 'Tissue-specific knockout of the insulin receptor in pancreatic beta cells creates an insulin secretory defect similar to that in type 2 diabetes', Cell, 96: 329-39.
Okada T, Liew CW, Hu J, Hinault C, Michael MD, Krutzfeldt J, Yin C, Holzenberger M, Stoffel M, Kulkarni RN. 2007. "Insulin receptors in {beta}-cells are critical for islet compensatory growth response to insulin resistance." In Proc Natl Acad Sci.
12
